# Atlas of exercise metabolism reveals time-dependent signatures of metabolic homeostasis

**DOI:** 10.1016/j.cmet.2021.12.016

**Published:** 2022-01-13

**Authors:** Shogo Sato, Kenneth A. Dyar, Jonas T. Treebak, Sara L. Jepsen, Amy M. Ehrlich, Stephen P. Ashcroft, Kajetan Trost, Thomas Kunzke, Verena M. Prade, Lewin Small, Astrid Linde Basse, Milena Schönke, Siwei Chen, Muntaha Samad, Pierre Baldi, Romain Barrès, Axel Walch, Thomas Moritz, Jens J. Holst, Dominik Lutter, Juleen R. Zierath, Paolo Sassone-Corsi

**Affiliations:** 1 Center for Epigenetics and Metabolism, INSERM U1233, Department of Biological Chemistry, School of Medicine, University of California, Irvine, Irvine, CA, USA; 2 Metabolic Physiology, Institute for Diabetes and Cancer, Helmholtz Diabetes Center, Helmholtz Zentrum München, German Research Center for Environmental Health, Neuherberg, Germany; 3 German Center for Diabetes Research, Neuherberg, Germany; 4 Novo Nordisk Foundation Center for Basic Metabolic Research, Faculty of Health and Medical Sciences, University of Copenhagen, Copenhagen, Denmark; 5 Department of Biomedical Sciences, Faculty of Health Science, University of Copenhagen, Copenhagen, Denmark; 6 Research Unit Analytical Pathology, Helmholtz Zentrum München, German Research Center for Environmental Health, Neuherberg, Germany; 7 Department of Molecular Medicine and Surgery, Integrative Physiology, Karolinska Institutet, Stockholm, Sweden; 8 Institute for Genomics and Bioinformatics, University of California, Irvine, Irvine, CA, USA; 9 Computational Discovery Research, Institute for Diabetes and Obesity, Helmholtz Diabetes Center, Helmholtz Zentrum München, German Research Center for Environmental Health, Neuherberg, Germany; 10 Department of Physiology and Pharmacology, Integrative Physiology, Karolinska Institutet, Stockholm, Sweden; 11 These authors contributed equally; 12 Present address: Center for Biological Clocks Research, Department of Biology, Texas A&M University, College Station, TX, USA; 13 Present address: Department of Medicine, Division of Endocrinology and Einthoven Laboratory for Environmental Vascular Medicine, Leiden University Medical Center, Leiden, the Netherlands; 14 Lead contact

## Abstract

Tissue sensitivity and response to exercise vary according to the time of day and alignment of circadian clocks, but the optimal exercise time to elicit a desired metabolic outcome is not fully defined. To understand how tissues independently and collectively respond to timed exercise, we applied a systems biology approach. We mapped and compared global metabolite responses of seven different mouse tissues and serum after an acute exercise bout performed at different times of the day. Comparative analyses of intra- and inter-tissue metabolite dynamics, including temporal profiling and blood sampling across liver and hindlimb muscles, uncovered an unbiased view of local and systemic metabolic responses to exercise unique to time of day. This comprehensive atlas of exercise metabolism provides clarity and physiological context regarding the production and distribution of canonical and novel time-dependent exerkine metabolites, such as 2-hydroxybutyrate (2-HB), and reveals insight into the health-promoting benefits of exercise on metabolism.

## In brief

Tissue sensitivity and response to exercise vary according to time of day. Sato et al. present an atlas of exercise metabolism, including global metabolomics profiling of multiple tissues, combined with arteriovenous sampling of hindlimb muscle and sampling across the liver to verify net uptake and release of time- and exercise-dependent signaling biochemicals.

## Graphical Abstract

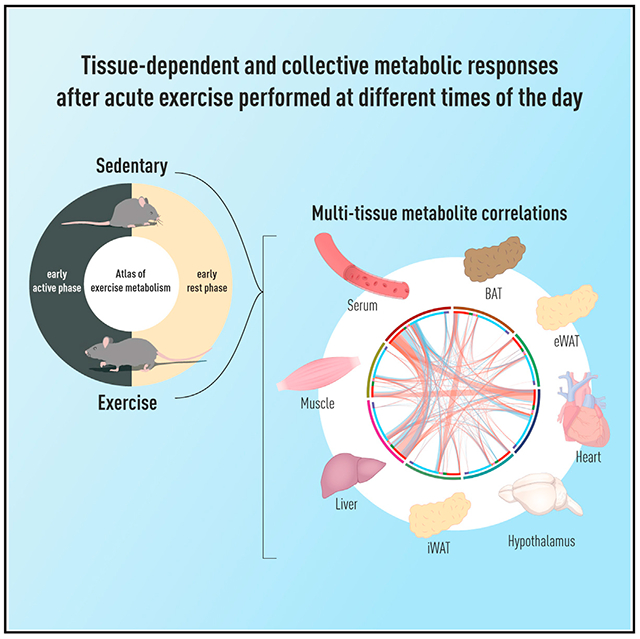

## INTRODUCTION

Circadian clocks orchestrate rhythmic biological processes, including metabolism, hormone production, immunity, and behavior. Temporal gating of physiology is essential for maintaining homeostasis, and chronic disruption of circadian alignment causes metabolic diseases (Masri and Sassone-Corsi, 2018). Light is the main *zeitgeber* (time giver) communicating external time to the central clock in the hypothalamic suprachiasmatic nucleus (SCN). Rhythmic feeding and locomotor activity, both SCN-controlled processes, entrain peripheral clocks in metabolic tissues independently of light (Damiola et al., 2000; Kemler et al., 2020; Small et al., 2020; Wolff and Esser, 2012). Timing of food intake can rewire temporal coordination of metabolism and gene expression and thereby modify disease progression (Hatori et al., 2012; Hawley et al., 2020; Lundell et al., 2020). Circadian control of systemic energy homeostasis and behavioral activity can also be entrained by exercise in a time-of-day-specific manner (Sato et al., 2019; Schroeder et al., 2012). Thus, food restriction, exercise, or energetic stressors that influence the temporal regulation of metabolism are relevant for glycemic control and weight loss in type 2 diabetes and obesity.

Exercise performed at different times of day influences skeletal muscle metabolic pathways and endurance capacity (Ezagouri et al., 2019; Sato et al., 2019). Thus, the timing of exercise may specify close alignment between tissue clocks and promote coherent and efficient temporal gating of metabolic processes. While the major effects of exercise on energy metabolism are well defined (Egan and Zierath, 2013; Hawley et al., 2014), a comprehensive view of how exercise timing determines tissue-specific metabolic adaptations and system-wide tissue coordination is lacking.

Here, we present an atlas of exercise metabolism, including global metabolomics profiling of multiple tissues and serum, and arteriovenous sampling of hindlimb muscle and sampling across the liver to verify net uptake and release of time- and exercise-dependent signaling biochemicals (exerkines). We show how timed exercise rewires tissue-specific and systemic metabolism and highlight differential tissue production and distribution of exerkines. We also validate 2-hydroxybutyrate (2-HB) as a time-dependent exerkine at the systemic level. Our resource provides physiological context about diurnal production and distribution of a wide range of signaling metabolites linked to sleep, memory, energy homeostasis, endurance capacity, and performance.

## RESULTS

### Time of exercise determines magnitude and type of metabolic response

**Figure 1.**
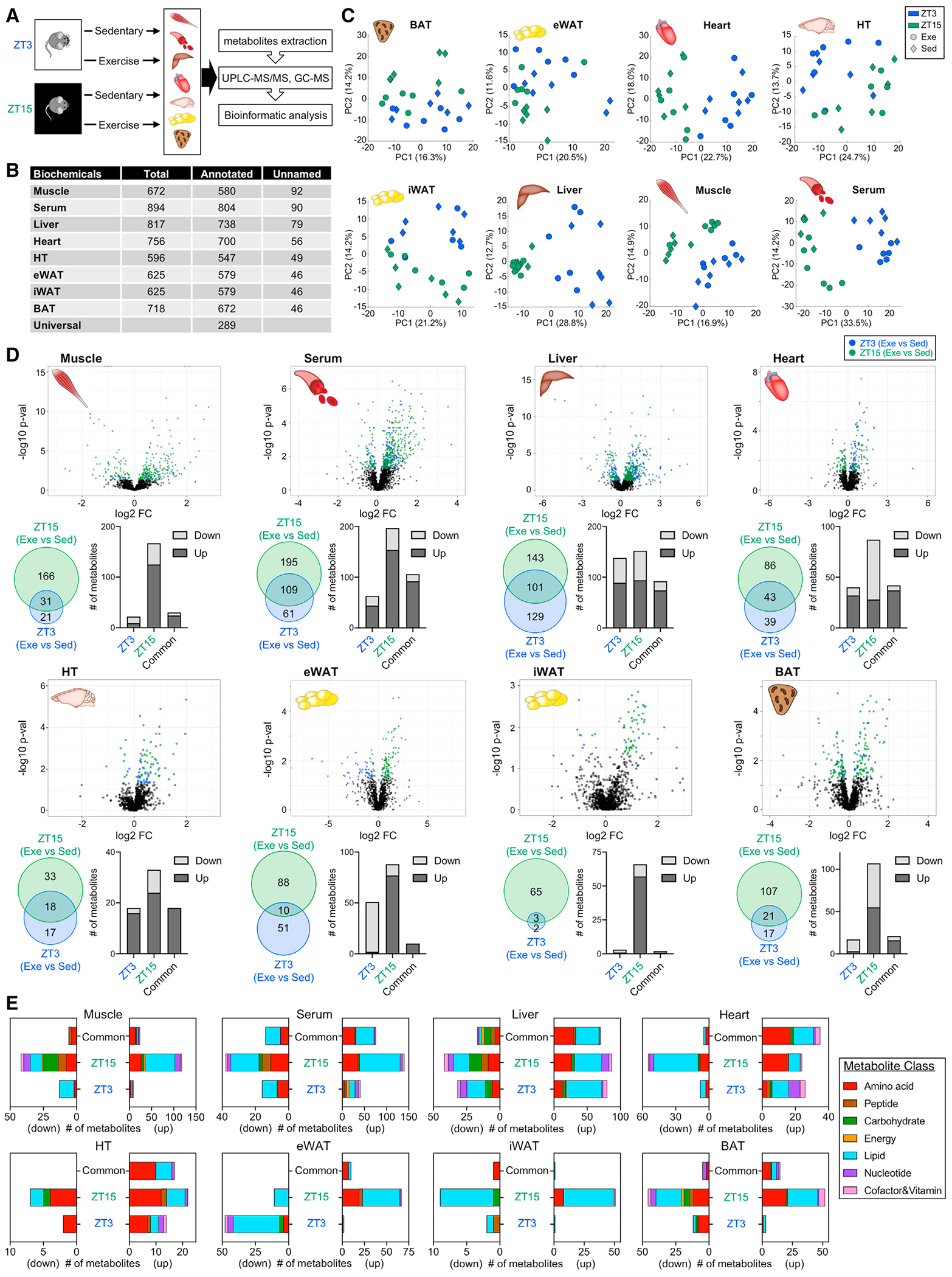
Multitissue metabolomics upon exercise at the early rest (ZT3) versus early active phase (ZT15) (A) Study design. Skeletal muscle, serum, liver, heart, hypothalamus (HT), epididymal white adipose tissue (eWAT), inguinal white adipose tissue (iWAT), and brown adipose tissue (BAT) were harvested after acute exercise at ZT3 or ZT15 for metabolomic analysis. (B) Detected biochemicals including named and unnamed biochemicals. (C) PCA plots of tissue-specific samples. Color of dots refers to time and shape refers to sedentary or exercise conditions. (D) Volcano plots, Venn diagrams, and bar charts of changed metabolites. (E) Biological classification of altered metabolites. n = 5–6 per group; altered metabolites determined by two-way ANOVA, p < 0.05. Sed, sedentary; Exe, exercise.

To determine how timing of exercise impacts local tissue and systemic metabolism, we collected serum, skeletal muscle (gastrocnemius), liver, heart, hypothalamus (HT), epididymal white adipose tissue (eWAT), inguinal subcutaneous white adipose tissue (iWAT), and interscapular brown adipose tissue (BAT). Exercise or control sham exercise was performed for 1 h on a treadmill at either ZT3 (early light/rest phase) or ZT15 (early dark/active phase), and tissues were collected at ZT4 or ZT16 immediately after exercise or control sham exercise, respectively (Figure 1A). We performed untargeted metabolomics and detected 600–900 metabolites in each tissue, including ~550–800 annotated metabolites (Figure 1B; Data S2). A common group of 289 annotated metabolites was detected in all tissues and serum (Figures 1B and S1A). PCA analysis revealed time (ZT) and treatment (exercise versus sedentary) effects on the metabolome for most of the tissues (Figures 1C and S1B). Each tissue displayed unique metabolic responses according to exercise time. Exercise at ZT15 altered 197 muscle metabolites, whereas 52 metabolites were impacted by exercise at ZT3 (Figures 1D, S1C, and S1D; Data S3). This included 31 metabolites common to both time points, and 166 (ZT15) and 21 (ZT3) metabolites specific to each. Serum, heart, HT, iWAT, and BAT showed the greatest response to exercise at ZT15. The liver displayed 129 and 143 metabolites impacted by exercise at ZT3 and ZT15, respectively, and 101 altered at both times. Altered eWAT metabolites were generally reduced by exercise at ZT3, yet most were increased by exercise at ZT15. Thus, exercise had a time-dependent and tissue-specific impact on metabolites.

Investigating these changes according to metabolite class (Figure 1E), amino acids (AAs) and lipids were more impacted by exercise at ZT15. However, the liver showed more lipids impacted by exercise at ZT3. Nucleotides were also more impacted by exercise at ZT15 in muscle, liver, and BAT, as were liver carbohydrates. A comparison of the top five metabolites altered in each tissue confirmed canonical exercise-associated changes and captured how the exercise responses largely reflect time-dependent differences in nutritional state and hormone production (Figure S1E). Corticosterone was a top-ranking metabolite increased by exercise at ZT3 in muscle, heart, HT, eWAT, iWAT, and BAT. Liver glycogen-derived metabolites maltopentaose, maltotetraose, and maltotriose showed the greatest reductions after exercise at ZT3, whereas glucose was decreased after exercise at ZT15. Lactate and indolelactate (indole-3-lactic acid), a tryptophan-derived lactate metabolite, were impacted by exercise at ZT15 in muscle, serum, heart, eWAT, and BAT. These results underscore how nutritional status changes over time and how exercise-dependent energy metabolism varies accordingly.

### Tissue- and time-dependent metabolomic effects of exercise

Corticosterone was increased by exercise in all tissues, but the liver and muscle showed higher levels after exercise at ZT3 (Figure S1F). Ketones like beta-hydroxybutyrate (BHB) and urea from ammonia detoxification were increased more by exercise at ZT15, indicating a greater reliance on fatty acid (FA) oxidation and increased buffering against metabolic stress during exercise performed in the early active phase (Figure S1F). Hypothalamic neurotransmitters serotonin, dopamine, and its catabolite homo-vanillate (HVA) increased by exercise at ZT15 (Figure S1G). Data were stratified to identify unique metabolites changing according to time of exercise and tissue (Data S4), allowing for the identification of tissue-specific biomarkers.

### Systemic activation of metabolic pathways in response to timed exercise

#### Functional enrichment of metabolites changed by exercise at different times of day

**Figure 2.**
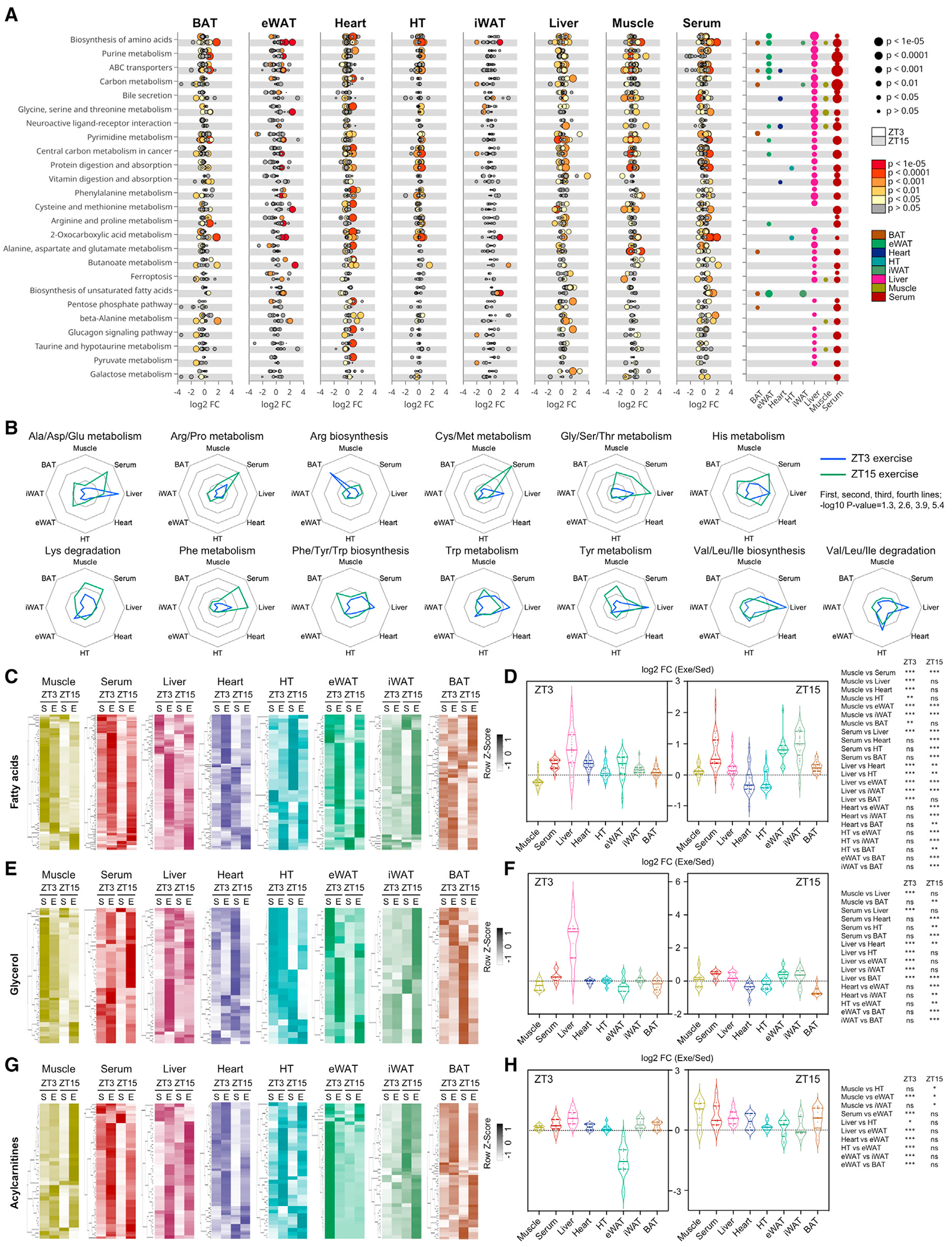
Spatiotemporal impacts of exercise on metabolism (A) KEGG enrichment analysis identifying time-dependent impact of exercise on local and systemic metabolism. Tissue-specific dotplots show regulation of metabolites mapped to specific pathways. Log_2_ FC (fold change) refers to exercise-induced metabolite regulation. Size and color of dot indicate significance of regulated (−log_10_(P)) metabolites. Gray dots refer to metabolites mapped to the pathway but not significantly regulated. Upper white and bottom gray areas indicate metabolites changed by exercise at ZT3 and exercise at ZT15, respectively. Significance of pathway enrichment is displayed on the right dotplot. Color of dots refers to tissue, and size refers to enrichment significance (−log_10_(p)). Significant (p < 0.001) pathways are shown. (B) Radar plots representing tissue-specific enrichment of regulated metabolites related to each amino acid class after exercise. First, second, third, and fourth lines from the center indicate −log_10_ p value = 1.3, 2.6, 3.9, and 5.4, respectively. (C) Heatmaps displaying metabolites related to fatty acids. (D) Effects of exercise at ZT3 (left) and ZT15 (right) on fatty acids common to tissues and serum. The y axis indicates log_2_ FC of levels of each fatty acid in exercise versus sedentary groups. (E) Heatmaps displaying metabolites related to glycerol. (F) Effects of exercise at ZT3 (left) and ZT15 (right) on glycerol common to tissues and serum. The y axis indicates log_2_ FC of level of each glycerol in exercise versus sedentary. (G) Heatmaps displaying metabolites related to acylcarnitines. (H) Effects of exercise at ZT3 (left) and ZT15 (right) on lipid oxidation common to the seven tissues and serum. The y axis indicates log_2_ FC of levels of each acylcarnitine in exercise versus sedentary. n = 5–6 per group; significantly altered metabolites determined by two-way ANOVA, p < 0.05. Sed, sedentary; Exe, exercise. One-way ANOVA to detect significance between tissues with Tukey post hoc testing.

To identify which metabolic pathways were directly impacted by exercise, orin a time- and tissue-specific manner, we performed Kyoto Encyclopedia of Genes and Genomes (KEGG) enrichment analysis on significantly altered metabolites (Figure 2A). Exercise at ZT15 elicited a robust enrichment of pathways involved in nucleotide metabolism (purine and pyrimidine) and AA metabolism (glycine, serine and threonine, phenylalanine, cysteine and methionine, arginine and proline, beta-alanine, and taurine and hypotaurine). Metabolites related to biosynthesis of unsaturated FAs were increased by exercise at ZT15 in both eWAT and iWAT. The liver showed distinct FA metabolism responses, with FA metabolites increased selectively by ZT3 exercise. This highlights a coregulation of lipid degradation pathways according to exercise time. Carbohydrate metabolism was increased in the liver during exercise at ZT3, indicating preferential utilization of glycogen. AA pathways were enriched in skeletal muscle by exercise at ZT15, including cysteine and methionine, along with purines. Serum exhibited the most striking response to exercise, preferably at ZT15, possibly because serum metabolites are integrative and systemic outcomes of the metabolic responses to exercise in each tissue.

#### Glycogenolysis and glycolysis

Muscle glycogen content was reduced after exercise at ZT15, but not ZT3 (Figure S2A), confirming our earlier findings (Sato et al., 2019). Hepatic glycogen content exhibited robust time-of-day variation. Accordingly, exercise at ZT3 decreased liver glycogen content, whereas at ZT15 glycogen stores were already low. Skeletal muscle and liver were the only tissues to show reduced glucose levels after exercise at ZT15 (Figure S2B). Glycolysis metabolites were also decreased after exercise at ZT15 in muscle but not in liver (Figure S2C), suggesting that during fasted conditions, exercise leads to a greater activation of muscle glycolysis with energy supplied from liver gluconeogenesis but not via hepatic glycogen breakdown. This was supported by gene expression profiles: muscle genes involved in glycolysis were increased after exercise at ZT15 (Figure S2D), whereas liver genes associated with gluconeogenesis were increased upon exercise at ZT3 (Figure S2E). Thus, glucose production from liver through glycogenolysis and gluconeogenesis may be sufficient during early rest phase exercise at ZT3. Glucose production from the liver may be suppressed during early active phase exercise at ZT15, leading to greater reliance on muscle glycogen.

#### AA metabolism

Since the KEGG enrichment pathway analysis indicated that AA metabolism exhibited a robust time-of-day-dependent impact of exercise, we explored tissue and time dependencies of exercise on AA metabolism. Heatmaps displayed global AA metabolites in tissues from sedentary and exercised mice (Figure S2F). AAs were highly enriched in serum of mice exercised at ZT15, suggesting increased protein degradation and AA utilization (Figure 2B). Muscle glycine, serine, threonine, lysine, and tyrosine metabolism were increased by exercise at ZT15, but not at ZT3. While levels of glycine, serine, threonine, and phenylalanine metabolism were also enriched after exercise at ZT15 in the liver, additional liver AAs exhibited enrichment after exercise at ZT3. Thus, hepatic AA may be produced and utilized differently according to exercise time.

#### Lipid metabolism

To understand how time of exercise modulates systemic lipid fluxes, we examined levels of metabolites related to FAs, glycerol, and acylcarnitines. Metabolites related to FA were extracted and displayed as heatmaps (Figure 2C), and the foldchange ratio of FA in exercise versus sedentary conditions at the corresponding time of day was compared (Figure 2D). There was a considerable induction of a subset of FAs in the liver by exercise at ZT3, whereas FAs in serum, eWAT, and iWAT were elevated by exercise at ZT15. Glycerol metabolites in the liver increased after ZT3 exercise, while the elevation was more prominent in iWAT after ZT15 exercise (Figures 2E and 2F). Thus, exercise at ZT3 stimulates lipolysis in liver, but exercise at ZT15 stimulates lipolysis in WAT. Acylcarnitine levels increased more upon ZT15 exercise in muscle and serum, suggesting activation of fat oxidation in muscle and higher energy demand from nonglycolytic sources by exercise at ZT15 (Figures 2G and 2H). These observations are supported by gene expression profiling: muscle genes involved in fat oxidation such as *Ppard* were upregulated exclusively after exercise at ZT15 (Figure S2G), while liver genes encoding lipases such as *Pnpla2* were elevated only after exercise at ZT3 (Figure S2H).

### Tissue specificity of metabolome response to exercise at different times of day

To understand which metabolic processes might be coupled according to time of exercise, we compared significantly altered metabolites between tissues. Pie charts showed only a marginal overlap (Figure S3A), indicating that exercise-induced metabolic alterations are tissue dependent. However, correlating metabolites commonly changed by exercise suggested more coordinated responses (Figure S3A). Upon exercise at ZT3, liver metabolites were less correlated with other tissues, whereas serum and BAT showed greater correlations with other tissues after exercise at ZT3. Muscle and liver metabolites were highly correlated upon exercise at ZT15 but attenuated after exercise at ZT3. The combination of eWAT and other tissues also exhibited a higher correlation coefficient at ZT15, pointing to a distinct metabolic response of eWAT according to exercise time. Metabolites commonly responding to exercise at ZT3 were more enriched with AAs than with lipids, while exercise at ZT15 increased enrichment of AAs and lipids. This highlights greater systemic coordination ofAA and lipid metabolism during ZT15 exercise. Carbohydrates were highly enriched within correlated metabolites between muscle and liver after ZT15 exercise. Thus, metabolic pathways are activated in a time- and tissue-dependent manner.

We then focused on metabolites changed within specific tissues or combinations of tissues (Figures S3B and S3C). After exercise at ZT3, the concentration of 14 metabolites (mostly lipids) was changed only in the liver and serum, while 6 AAs unique to serum and heart were changed. The correlation of metabolites altered within serum and liver and within serum and heart was significant (Figure S3D). In response to exercise at ZT15, several metabolites were altered that were unique to serum, liver, or muscle (Figure S3C), with a group of carbohydrates enriched the most. Distinct clusters related to glycogen and glucose metabolism were among metabolites changed between muscle and liver after exercise at ZT15 (Figure S3E), pointing to the activation of glycogenolysis and glycolysis as a common metabolic hallmark. Metabolites commonly changed in serum and tissues were predominately lipids and AAs (Figure S3C), including a common group of lipids in serum and iWAT after ZT15 exercise (Figure S3E). We found systemic and more coordinated activation of lipid and AA metabolism, as well as local activation of carbohydrate metabolism specific to muscle and liver after exercise in the fasted state.

### Exercise rewires intra-tissue and inter-tissue metabolite correlations

**Figure 3.**
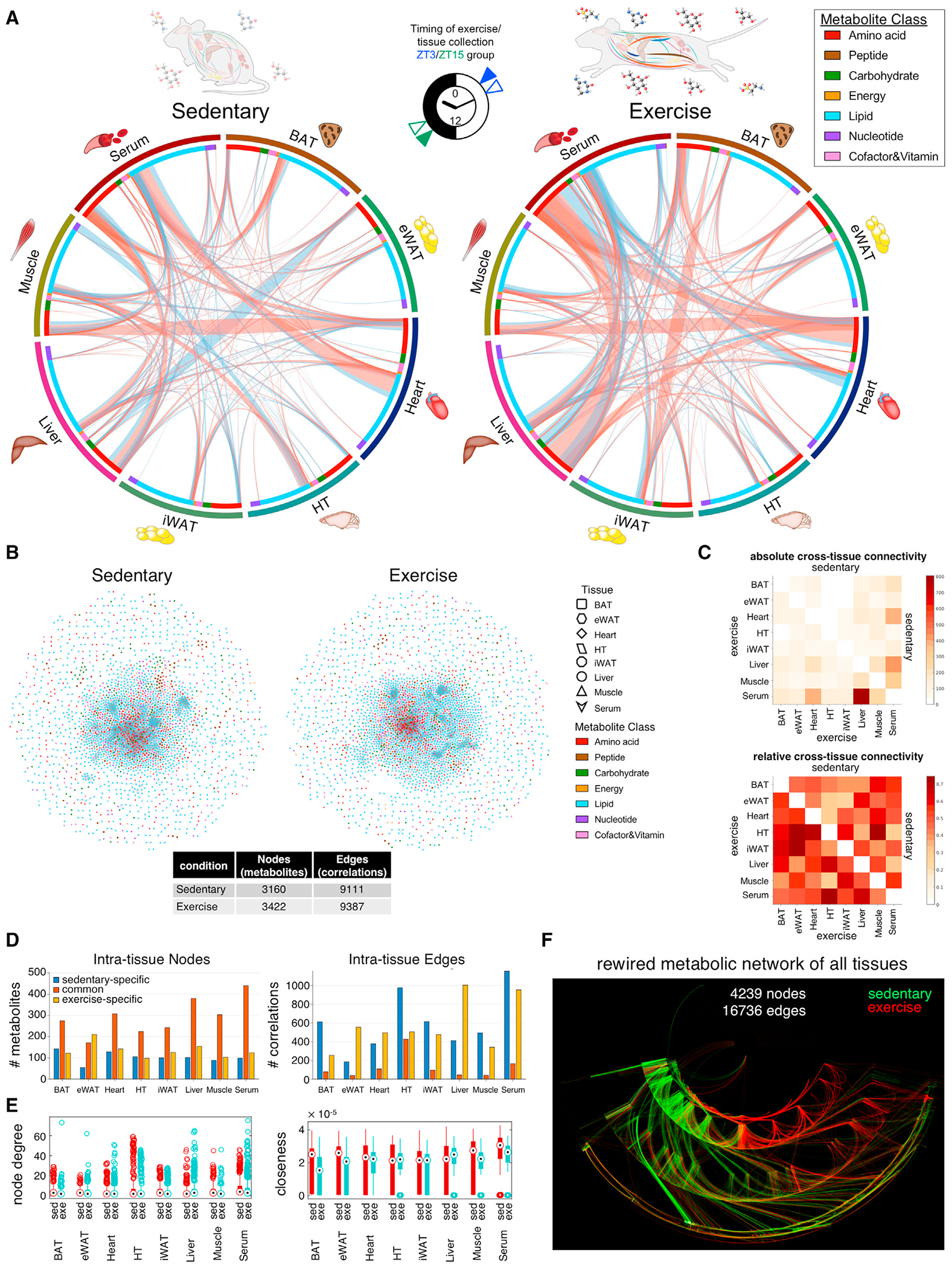
Exercise rewires intra- and inter-tissue metabolic correlations (A) Summary of inter-tissue metabolite correlations. Colored circles refer to tissue and metabolite classes. Ribbons connecting metabolite inter-tissue classes refer to significant correlations between metabolites. Ribbon color refers to correlation coefficient sign (red, positive; blue, negative). Ribbon thickness refers to number of significantly correlated metabolites. The clock icon indicates the timing of exercise (filled triangles) and tissue collection (opened triangles). (B) Networks of significantly correlated metabolites. Each node refers to a metabolite, shape indicates tissue, and color refers to metabolite class. Edges are drawn for each intra-tissue or inter-tissue correlation, and color refers to correlation coefficient sign (red, positive; blue, negative). Node and edge numbers are summarized in the table. (C) Heatmaps show absolute (upper) and relative (lower) cross-tissue connections (significant correlations). Upper right triangles refer to sedentary conditions, and lower left triangles refer to exercise conditions. (D) Number of common, sedentary-, or exercise-dependent nodes and edges associated with each tissue. (E) Boxplot of network properties according to tissue. Node degree refers to number of connections of each node, and closeness refers to closeness centrality. Black dots display the median, the size of the boxes the upper and lower quartiles, whiskers minimum and maximum values, and circles refer to outliers. (F) Combined DyNet visualization of all tissues and serum with a metabolite interaction network highlighting rewired nodes and edges. Red nodes and edges are present only under exercise; green nodes and edges are present only under sedentary conditions; gray nodes and edges are present under both.

Mapping all pair-wise metabolite correlations (Figure 3A), exercise mainly enhanced interactions among serum, heart, liver, and muscle. These differences reflect broad exercise-dependent shifts in tissue coordination in support of energy substrate supply and demand. To reveal underlying metabolic relationships, we combined all tissues and constructed undirected condition-specific correlation networks (Figure 3B). Sedentary and exercise groups shared a similar number of nodes (~3,000 metabolites correlated across tissues) and edges (~9,000 correlations) (Figure 3B). More cross-tissue correlations were found after exercise (Figure 3C). Examining intra-tissue correlations, ~50%–60% nodes were common to sedentary and exercise groups (eWAT as the exception), while only ~20% were specific to either condition (Figures 3D and S4A–S4D; Data S5). Edges reflected exercise-induced metabolic rewiring, with few shared connections between sedentary and exercise conditions. Exercise reprogrammed interactions and reduced overall connectivity within BAT and HT, whereas muscle, heart, iWAT, and serum retained connectivity. Metabolite connectivity was increased within the liver and eWAT, underscoring its prominent role as a metabolic hub; liver node “degree distribution” (edges per node) and “closeness distribution” (centrality of nodes corresponding to the whole network) were increased by exercise (Figure 3E). Integrating networks into a metabolite “correlation universe” revealed how networks within and among tissues are rewired by exercise. Nodes with the highest connectivity were major points of diversion among conditions (Figures 3F and S4D). Inspection of the top commonly reprogrammed nodes revealed that exercise increased connectivity among metabolites involved in AA and lipid metabolism. These include *N*-acetylleucine, 2-HB/2-hydroxyisobutyrate, ketone body BHB, carnitine synthesis metabolite hydroxy-*N*6,*N*6,*N*6-trimethyllysine, the uremic toxin phenol sulfate, and the purine nucleotide guanine. Exercise reduced connectivity of the tryptophan catabolite and neurotoxin quinolinate, as well as *N*-acetylglycine, and a variety of FAs and glycerophospholipids (Figures S4E and S4F).

### Exercise at ZT15 selectively strengthens skeletal muscle-liver 24-h tissue correlations

To investigate how time and exercise interact to determine intra-tissue and inter-tissue metabolite temporal correlations, we focused on muscle and the liver, major organs involved in energy homeostasis during exercise and food restriction. We calculated pair-wise metabolite temporal correlations within and between tissues collected at 4-h intervals across 24 h after an exercise bout performed at ZT3 or ZT15 versus sedentary controls (Figures S5A and S5B). Exercise timing determined the number (Figures S5C–S5F), amplitude (Figure S5G), phase (Figures S5H and S5I), and class (Figures S5J and S5K) of 24-h oscillating liver metabolites (Data S2 and S6). Comparing 24-h metabolomes further revealed tissue-, time-, and exercise-dependent alterations (Figure S5L).

**Figure 4.**
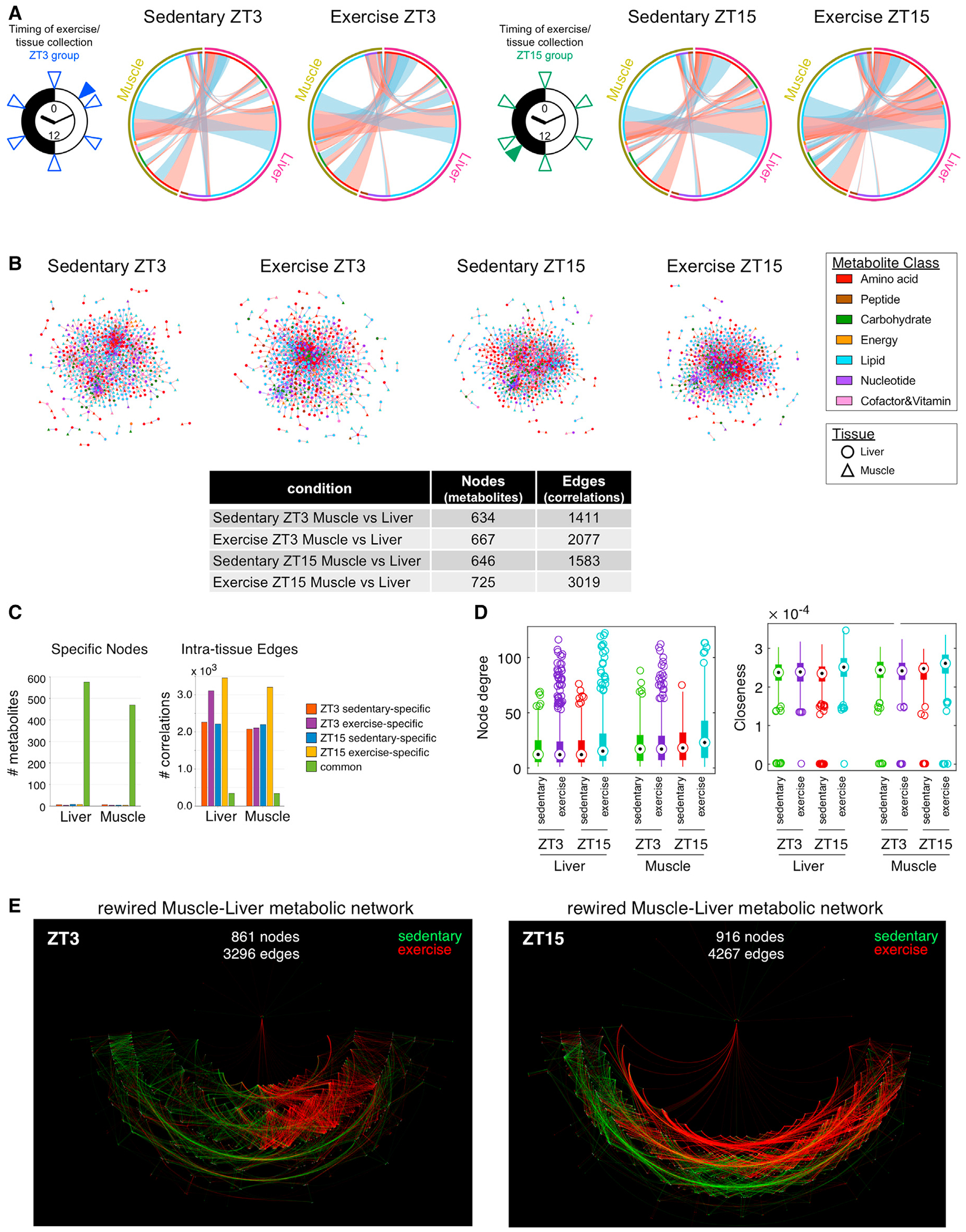
Exercise time determines muscle-liver 24-h temporal correlation (A) Muscle-liver metabolite correlations according to condition and time. Colored circles refer to tissue and metabolite class. Ribbons refer to correlations between metabolites. Ribbon color refers to correlation coefficient sign (red, positive; blue, negative). Ribbon thickness refers to number of correlated metabolites. Clock icons indicate the time of exercise (filled triangles) and tissue collection (opened triangles) during ZT3 and ZT15. (B) Networks of muscle-liver correlated metabolites. Each node refers to a metabolite, shape indicates tissue, and color refers to metabolite class. Edges are drawn for each intra-tissue or inter-tissue correlation, and color refers to correlation coefficient sign (red, positive; blue, negative). Table shows total number of nodes (metabolites) and edges (significant correlations) in muscle and liver according to condition and time. (C) Number of common, sedentary-, or exercise-dependent nodes and edges at each time point between muscle and liver. (D) Boxplots of network properties according to tissue, time, and condition. Node degree refers to the number of connections of each node, and closeness refers to closeness centrality. Black dots display the median, the size of the boxes the upper and lower quartiles, whiskers minimum and maximum values, and circles refer to outliers. (E) Combined DyNet visualization of muscle-liver metabolite interaction network highlighting rewired nodes and edges. Red nodes and edges are present only under exercise; green nodes and edges are present only under sedentary conditions; gray nodes and edges are present under both.

The liver and muscle shared fewer correlations under sedentary conditions, with ZT3 sharing the fewest (Figures 4A and 4B; Data S6). Exercise increased temporal correlations between lipids, AAs, nucleotides, and carbohydrates, but the effect was greater at ZT15. Constructing 24-h correlation networks for each condition (Figure 4B), we visualized how exercise increased overall metabolite connectivity between tissues in a time-dependent manner. We detected a similar range of ~600–700 correlated metabolites under sedentary and exercise conditions. Edges were highly context dependent, with 1,411 (ZT3) and 1,583 (ZT15) temporal correlations detected under sedentary conditions, and 2,077 (ZT3) and 3,019 (ZT15) detected after exercise. Most nodes were common between conditions, and yet, the edges were highly context dependent (Figure 4C). Node degree and closeness were increased by exercise at ZT15 (Figure 4D), highlighting distinct timing differences in metabolite response and network wiring (Figure 4E). Our data reveal how intra-tissue and inter-tissue metabolism are differentially poised to both react and respond to exercise at different times of the day.

### Time and tissue dependency of exercise-induced metabolites

**Figure 5.**
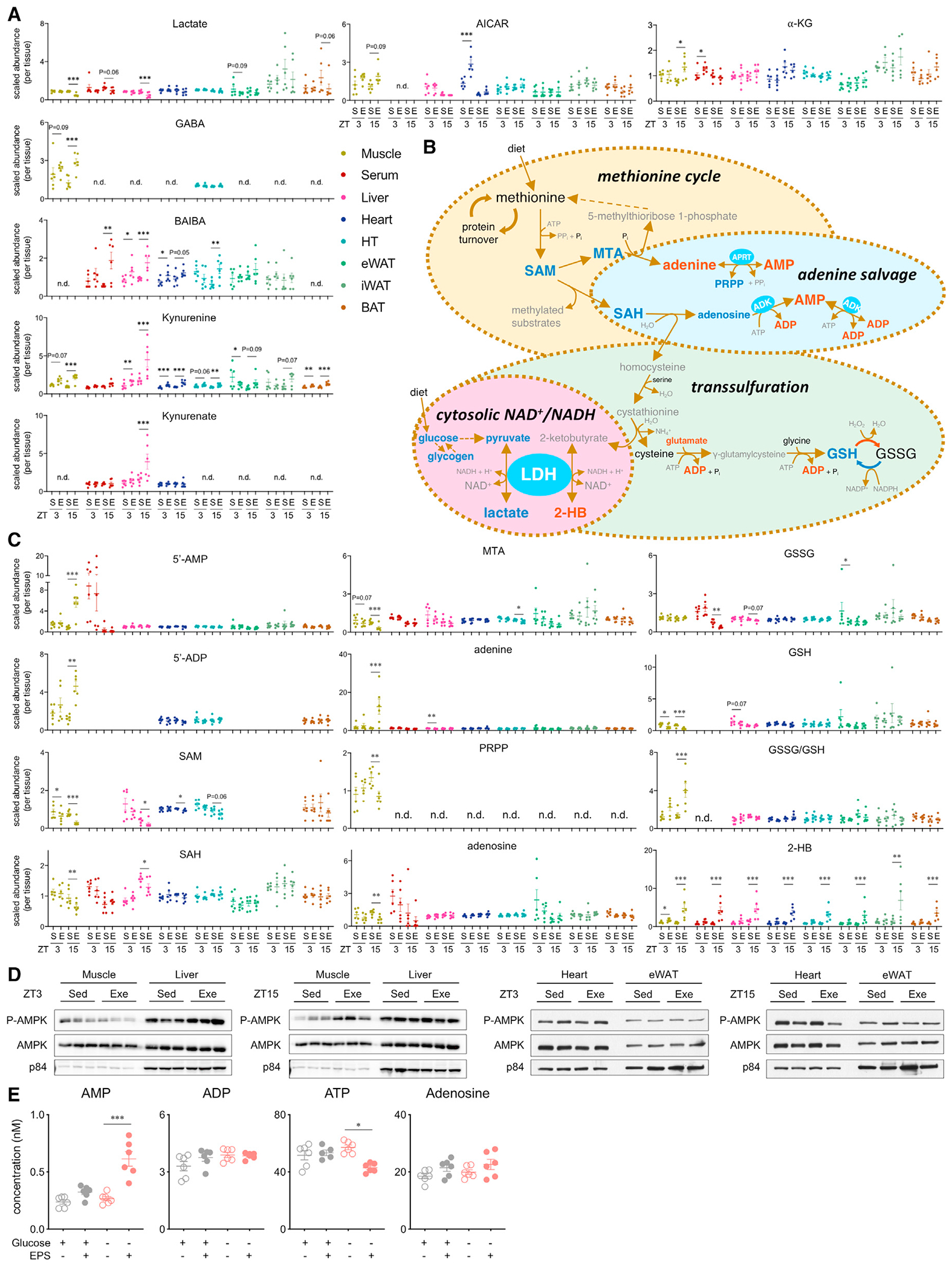
Tissue- and time-dependent links between SAM metabolism, adenine salvage, GSH production, and maintenance of cytosolic NAD^+^/NADH (A) Exerkine metabolite plots. Individual data points are colored according to tissue and represented as mean ± SEM (n = 5–6) and analyzed by two-way ANOVA (sedentary versus exercise at corresponding time), *p < 0.05, **p < 0.01, and ***p < 0.001. GABA, gamma-aminobutyrate; BAIBA, 3-aminoisobutyrate; AICAR, 5-aminoimidazole-4-carboxamide ribonucleotide; α-KG, α-ketoglutarate. (B) Pathway scheme of methionine cycle, purine salvage, and transsulfuration pathways. Blue metabolites, reduced in muscle by exercise at ZT15; orange metabolites, increased in muscle by exercise at ZT15; black metabolites, unchanged; gray metabolites, no data. (C) Metabolite plots of SAM metabolism in relation to methionine cycle, purine salvage, and transsulfuration pathways. Individual data points are colored according to tissue and represented as mean ± SEM (n = 5–6) and analyzed by two-way ANOVA (sedentary versus exercise at corresponding time), *p < 0.05, **p < 0.01, and ***p < 0.001. SAM, *S*-adenosylmethionine; MTA, 5-methylthioadenosine; SAH, *S*-adenosylhomocysteine; PRPP, 5-phosphoribosyl diphosphate; AMP, adenosine 5’-monophosphate; APRT, adenine phosphoribosyltransferase; ADK, adenosine kinase; AK, adenylate kinase; ADP, adenosine 5’-diphosphate; ATP, adenosine triphosphate; GSH, reduced glutathione; GSSG, oxidized glutathione; LDH, lactate dehydrogenase; 2-HB, 2-hydroxybutyrate; n.d., no data. S and Sed, sedentary; E and Exe, exercise. (D) AMPK protein and phosphorylation in tissues. (E) AMP, ADP, ATP, and adenosine concentration in myotubes. Individual data points are represented as mean ± SEM (n = 6) and analyzed by two-way ANOVA using Bonferroni post hoc testing (with versus without EPS), *p < 0.05 and ***p < 0.001.

Our resource allows for comparison of metabolite exerkines and provides context about their production and distribution in a time- and tissue-dependent manner. We noted distinct baseline, exercise time-, and tissue-dependent differences among established exerkines, including lactate, gamma-aminobutyrate (GABA), 3-aminoisobutyrate (BAIBA), kynurenine, kynurenate, 5-aminoimidazole-4-carboxamide ribonucleotide (AICAR), and α-ketoglutarate (α-KG) (Figure 5A), with greater responses at ZT15. Lactate was reduced, whereas α-KG and kynurenine were increased in muscle and the liver at ZT15. Kynurenate was increased by exercise only in liver at ZT15. BAIBA was increased by exercise in serum, liver, heart, and HT at ZT15. GABA was detected in muscle and HT but was increased by exercise only in muscle. AICAR was increased more by exercise at ZT3 in the heart, as was serum α-KG.

Exercise at ZT15 appeared to preferentially direct skeletal muscle *S*-adenosylmethionine (SAM) metabolism toward adenine nucleotide salvage, transsulfuration pathways linked to glutathione (GSH) production, and maintenance of cytosolic NAD^+^/NADH (Figures 5B and 5C). Exercise at ZT15 reduced SAM levels in muscle, liver, and heart. In muscle, exercise reduced downstream SAM metabolites *S*-adenosylhomocysteine (SAH), 5-methylthioadenosine (MTA), adenosine, and GSH, while the ratio of oxidized to reduced glutathione (GSSG/GSH) was increased at ZT15. Muscle adenine and 5′-AMP were increased by exercise only at ZT15. Adenine conversion to AMP can be increased by availability of 5-phosphoribosyl-1-pyrophosphate (PRPP), which at ZT15 was increased in muscle under sedentary conditions and reduced by exercise. We confirmed the specificity of this metabolic signal by immunoblot analysis of activated AMPK (Figure 5D). Skeletal muscle contains a large pool of adenine nucleotides and maintaining sufficient ATP levels is important to sustain contractions. To avoid metabolic stress and fatigue, adenine nucleotide degradation must be balanced by increased salvage and *de novo* synthesis (Brault and Terjung, 2001). Our results suggest exercise-induced adenine nucleotide salvage is regulated locally within the muscle in a time-dependent manner.

To further clarify the time-dependent activation of skeletal muscle AMPK upon exercise, we determined how the nutritional state influences adenine nucleotide metabolism. To mimic exercise under different nutritional states, myotubes were subjected to electrical pulse stimulation (EPS) in the absence or presence of glucose. We measured adenine nucleotides and adenosine using liquid chromatography-mass spectrometry (LC-MS) (Figure 5E). AMP production was elevated by EPS in glucose-depleted conditions, consistent with our finding that exercise at ZT15 increased muscle AMP levels and AMPK activation (Figures 5C and 5D). ATP regeneration was attenuated by EPS in glucose-depleted conditions.

Liver and skeletal muscle are producers and net exporters of circulating GSH (Kretzschmar and Muller, 1993; Sen, 1998). GSH production depends on cysteine generated from transsulfuration of homocysteine, with increased flux shown to delay ageing and increase lifespan (Sbodio et al., 2019). SAH produced from SAM-mediated methylation reactions is rapidly converted to homocysteine, which, in the presence of sufficient methionine and vitamin B6, proceeds via cystathionine toward the formation of 2-ketobutyrate (2 KB; also known as 2-oxobuty-rate), cysteine, and ammonia (Figures 5B and 5C). As a structural analog of pyruvate, 2-KB is readily reduced to 2-HB by lactate dehydrogenase, causing regeneration of NAD^+^. Since this is a near-equilibrium reaction (Rogatzki et al., 2015), exercise-induced accumulation of 2-HB likely reflects a combination of factors, including cytosolic redox imbalance and energy stress. 2-HB was proposed as a specific *in vivo* biomarker for NAD^+^/NADH ratio in liver (Goodman et al., 2020), but since it showed time-dependent accumulation in all tissues studied, it may be a general indicator of systemic NAD^+^/NADH.

### Time-of-day- and exercise-dependent arteriovenous balance of metabolites

**Figure 6.**
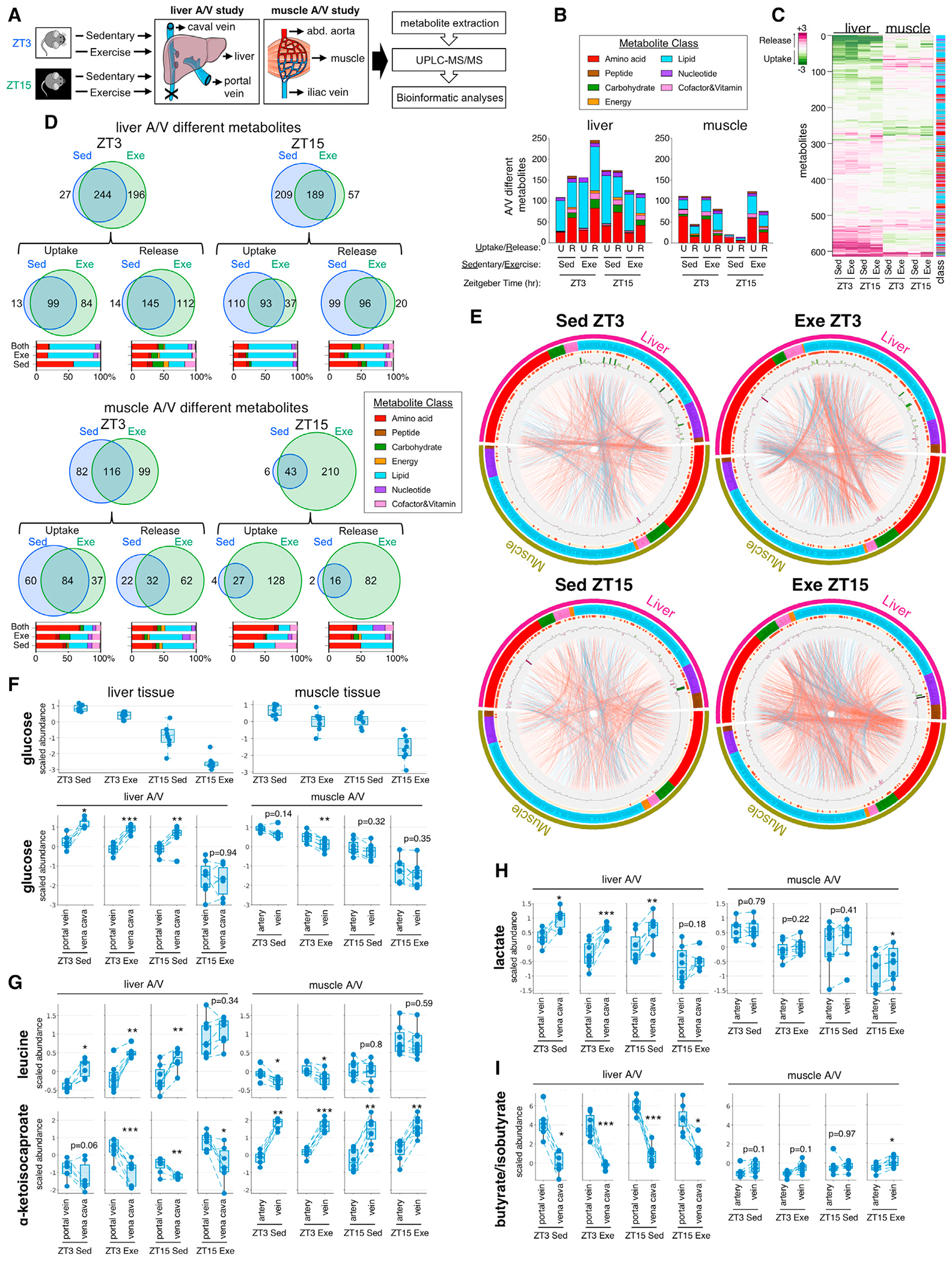
Arteriovenous (A/V) differences of liver and muscle in response to timed exercise (A) Study design. Tissues were collected immediately after exercise at ZT3 or ZT15. For sampling across the liver, serum was collected from the portal vein and caval vein. For hindlimb muscle A/V sampling, serum was collected from the abdominal aorta (abd. aorta) and iliac vein. Figure elements modified from SMART (Servier Medical Art), licensed under a Creative Common Attribution 3.0 Generic License. http://smart.servier.com/. (B) Number and class of altered A/V metabolites (adjusted p < 0.05, paired t test). (C) Heatmap displaying A/V metabolite dynamics. (D) Venn diagrams and relative class distribution of altered A/V metabolites (adjusted p < 0.05, paired t test). (E) Muscle-liver metabolite correlations according to condition, time, and relative tissue uptake/release. Outer ring designates tissue. Second inner ring is colored according to metabolite class. Red bands on the third inner ring designate altered A/V metabolites (adjusted p < 0.05, paired t test), while the innermost ring designates relative tissue uptake (green) or release (purple) for each metabolite. Ribbons connecting metabolite inter-tissue classes refer to correlations between metabolites from Figure 4A. Ribbon color refers to correlation coefficient sign (red, positive; blue, negative). Each link refers to a correlated metabolite pair. (F–I) Boxplots and relative A/V differences of selected metabolites (n = 6; adjusted *p < 0.05, **p < 0.01, and ***p < 0.001, paired t test; horizontal lines of boxes indicate the median, size of the boxes reflects upper and lower quartiles, whiskers are minimum and maximum values, and dashed lines connect A/V data from individual mice).

To gain insight into dynamic metabolite exchange between tissues, we exercised mice at different times of the day and profiled arteriovenous differences in metabolites across hindlimb muscles and the liver (portal to hepatic vein). We collected (1) serum from the portal vein and vena cava (mainly liver veins) and liver and (2) arterial and venous serum and skeletal muscle (Figure 6A).

We detected hundreds of metabolites and related pathways with time- and exercise-dependent arteriovenous (A/V) differences in each tissue (Figures 6B–6D, S6A, and S6B; Data S2). At ZT3, the liver was a net exporter of AAs, lipids, nucleotides, carbohydrates, TCA (tricarboxylic acid) cycle intermediates, and a variety of vitamins and cofactors, and an importer of lipids, AAs, and nucleotides (Figure 6B). Liver uptake and release were increased by exercise at ZT3 but reduced at ZT15. Muscle was a net importer of AAs, lipids, carbohydrates, nucleotides, and other energy metabolites while releasing ~20%–50% fewer metabolites than consumed. Net changes in muscle metabolite balance were minimal under sedentary conditions at ZT15, and yet, with exercise, the net metabolite uptake increased to ZT3 levels. Exercise increased net muscle metabolite release at both time points. This likely reflects time- and exercise-associated changes in blood flow (Bartman et al., 2021) and highlights the importance of physical activity in the production and release of muscle-derived biomolecules.

Visualizing all A/V muscle and liver metabolites with correlations derived from tissue metabolite profiling (Figure 4A) revealed the direction of metabolite flow between tissues in relation to coupled metabolic processes (Figures 6E and S6B). Increased correlations after exercise at ZT3 were driven largely by metabolite exchanges between muscle and liver. Increased correlations after exercise at ZT15 reflected a coordinated release of metabolites by muscle and liver. Liver and muscle were the only tissues to show reduced glucose concentrations after exercise at ZT15 (Figure S2B), with liver glucose also reduced under sedentary conditions. Thus, we hypothesized that after exercise at ZT15, liver glucose production and release were insufficient to maintain glucose levels and meet increased energetic demands. A/V data confirmed that systemic glucose was reduced after exercise at ZT15 (Figure 6F). Net liver glucose release was blunted only after exercise at ZT15. Net muscle glucose uptake was increased only upon exercise at ZT3. This inter-tissue glucose exchange supports intra-tissue glucose and glycogen dynamics and subsequent carbohydrate utilization in response to exercise in a time-dependent fashion.

The branched-chain amino acids (BCAAs) leucine, isoleucine, and valine showed net release by the liver under sedentary and exercise conditions at ZT3 and under sedentary conditions at ZT15. Only isoleucine showed net release by liver after exercise at ZT15 (Figures 6G, S6C, and S6D). BCAAs were similarly taken up by muscle under sedentary and exercise conditions at ZT3. Circulating BCAAs were highest after exercise at ZT15, but the net BCAA balance in muscle did not change. Branched-chain keto acids α-ketoisocaproate (4-methyl-2-oxopentanoate), α-keto-β-methylvalerate (3-methyl-2-oxovalerate), and α-keto-isovalerate (3-methyl-2-oxobutyrate) showed net release by muscle and net uptake by the liver, regardless of the time of day or exercise.

Contextualizing A/V differences for the range of metabolites known to increase after exercise (Morville et al., 2020), one can reconstruct relative changes in metabolic wiring and infer the direction of flow. The liver showed net lactate release under all conditions except for exercise at ZT15, whereas muscle showed net lactate release only after exercise at ZT15 (Figure 6H). TCA cycle intermediates showed heterogeneous responses (Figure S6E). α-KG showed net uptake by the liver under all conditions and time points. While α-KG trended toward net muscle release, this was significant only at ZT3. Succinate showed net liver release under all conditions, and yet, the only significant change in muscle was the net uptake after exercise at ZT15. Fumarate and malate showed similar trends of net release by the liver, but release by the muscle was only significant after exercise at ZT15. These changes reflect a flexible, dynamic, and context-specific flux into central metabolic pathways at different entry and exit points.

We noted time- and exercise-dependent A/V differences for metabolites associated with healthy aging (Figure S6F). Liver and muscle were both net exporters of the NAD^+^ precursor nicotinate riboside. While liver release was significant under exercise and sedentary conditions, muscle release was significant only after exercise. Net liver release of spermidine was significant only under sedentary conditions, whereas net muscle release was significant only after exercise.

A/V data also revealed regional differences in circulating metabolite concentrations, with relatively high concentrations of portal vein short-chain FAs like butyrate/isobutyrate, which are produced mainly in the colon by bacterial fermentation. The highest butyrate/isobutyrate levels were detected in the portal vein at ZT15 in sedentary mice (Figure 6I), with low net export from the liver under all conditions. Muscle was a net exporter of butyrate/isobutyrate after exercise at ZT15, although relative concentrations were much lower than that in the portal vein.

### Time-of-day-dependent role of 2-HB in systemic and local tissue metabolism

**Figure 7.**
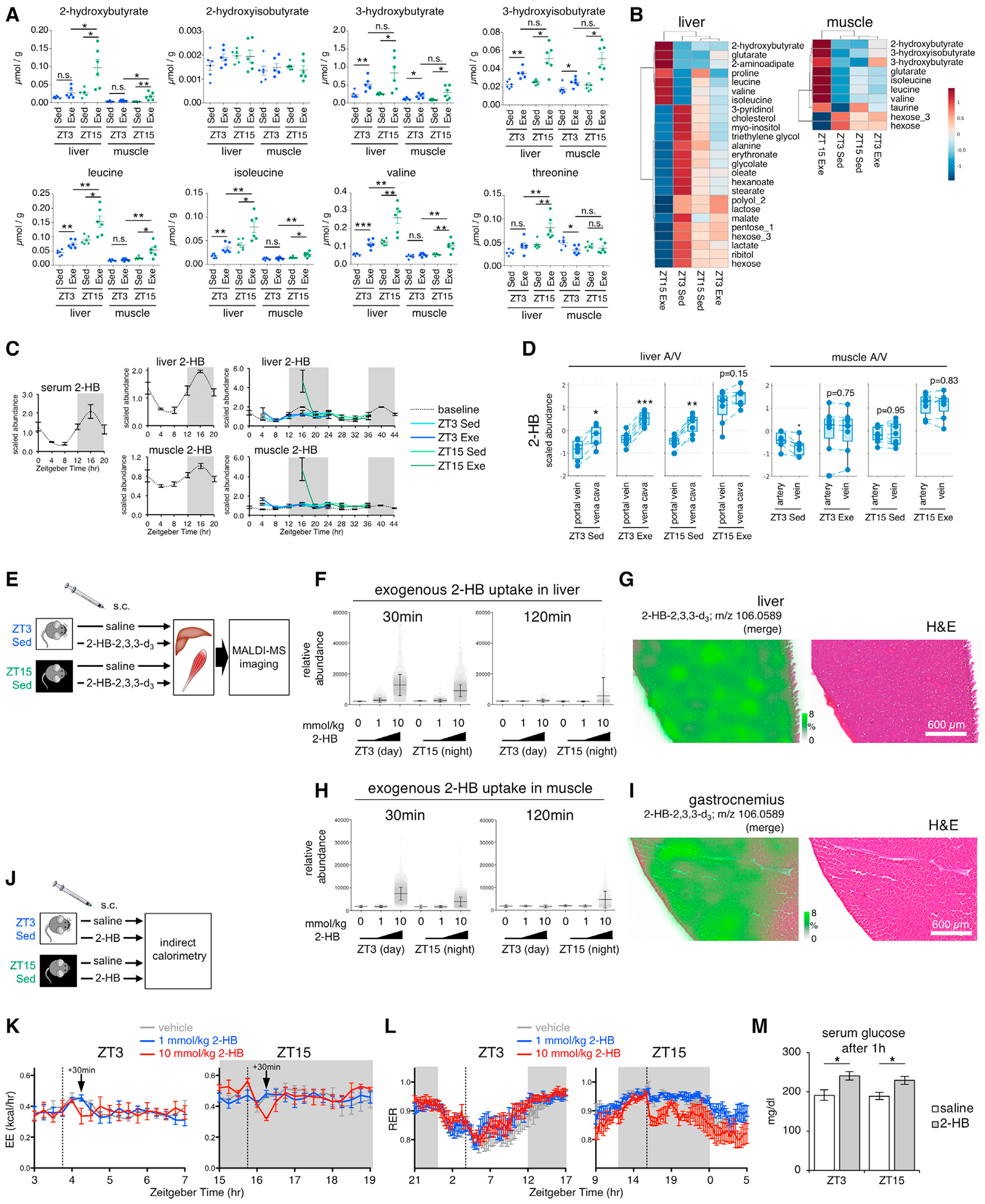
Validation of 2-hydroxybutyrate (2-HB) as a time-dependent exerkine (A) Quantification of selected metabolites by targeted gas chromatography mass spectrometry using spiked internal standards (n = 6; n.s., not significant, *p < 0.05, **p < 0.01, and ***p < 0.001, unpaired two-tailed t test). (B) Heatmap of targeted metabolites showing top 25 (liver) and 10 (muscle) most significant features detected by PCA plot. (C) Diurnal variations of 2-hydroxybutyrate (2-HB) (mean ± SEM; n = 5–6; baseline data derived from Dyar et al. (2018b). (D) Boxplots and relative A/V differences of 2-HB (n = 6; adjusted *p < 0.05, **p < 0.01, and ***p < 0.001, paired t test; horizontal lines of boxes indicate median, size of boxes reflects upper and lower quartiles, whiskers are minimum and maximum values, and dashed lines connect A/V data from individual mice). (E) Study design for MALDI-MS imaging validation using deuterated 2-HB. s.c., subcutaneous. (F) Quantification of exogenous 2-HB (mean pixel intensity ± SEM). (G) Liver section showing exogenous 2-HB 30 min after injection (10 mmol/kg) at ZT15. (H) Quantification of exogenous 2-HB (mean pixel intensity ± SEM). (I) Muscle section (gastrocnemius) showing exogenous 2-HB 30 min after injection (10 mmol/kg) at ZT3. (J) Study design for indirect calorimetry using unlabeled 2-HB. s.c., subcutaneous. (K and L) Effects of timed 2-HB administration on energy expenditure (EE, K) and respiratory exchange ratio (RER, L) measured at 23°C. n = 4–5; mean ± SEM. (M) Effects of timed 2-HB administration (10 mmol/kg) on glycemia (mean ± SEM; n = 4–6; *p < 0.05, unpaired two-tailed t test).

We selected 2-HB/2-hydroxyisobutyrate for further investigation since this metabolite emerged as a central systemic metabolic hub, connecting metabolites in a time- and exercise-dependent fashion (Figure S4E). 2-HB and 2-hydroxyisobutyrate (2-HIB) are isobaric isomers. To clarify this signal and to quantitatively validate additional hits, we used targeted gas chromatography-mass spectrometry (GC-MS) with spiked internal standards. Endogenous 2-HB was elevated in the liver and muscle following exercise at ZT15 (Figure 7A). The liver showed higher levels (~0.01–0.2 μmol/g) than muscle (~0.002–0.03 μmol/g). These concentrations and dynamics resembled AAs, including BCAAs and threonine. The ketone body BHB showed similar dynamics, albeit at 103 higher levels. 2-HIB was unaltered in response to time or exercise, and concentrations remained near the limit of detection (LOD) and ~203 lower than 2-HB. Thus, exercise increased the production of 2-HB in a time-dependent manner. Applying unsupervised clustering of targeted metabolomics data (Figure 7B), 2-HB was highly correlated with other hydroxybutyric acids, and biomarkers for diabetes (Newgard et al., 2009; Nilsen et al., 2020; Wang et al., 2013), yet inversely correlated with hexoses.

To understand how temporal 2-HB dynamics are impacted by exercise (Figure 7C), we compared baseline data from sedentary mice (Dyar et al., 2018b) to our data after timed exercise. 2-HB levels in serum, liver, and muscle oscillate together over 24 h, with lower levels during the light phase and ~2- to 4-fold higher levels during the dark phase. Exercise and sham exercise elevated 2-HB levels in liver and muscle during trough levels at ZT4, but the increase was ~23 greater with exercise. Exercise at ZT15 increased 2-HB concentration ~3–53 beyond the normal peak, but levels returned to baseline within 4 h. To gain insight into relative tissue 2-HB uptake and release, we examined our A/V data. Net release by liver remained significant under all conditions, except for ZT15 exercise, when systemic 2-HB levels were highest (Figure 7D). Muscle net uptake of 2-HB was significant only under ZT3 sedentary conditions, when systemic levels were lowest.

To investigate the role of 2-HB as a signaling molecule, we administered deuterated 2-HB to mice at different doses and times of day (Figure 7E). We collected tissues and monitored 2-HB biodistribution and its impact on metabolism using matrix-assisted laser desorption/ionization mass spectrometry (MALDI-MS) imaging (Kunzke et al., 2020; Prade et al., 2020). Exogenous 2-HB (*m*/*z* 106.0589) was detected in liver and muscle in a dose- and time-dependent manner (Figures 7F–7I, S7A, and S7B). Liver and muscle showed similar uptake of 2-HB regardless of the time of day, but exogenous 2-HB remained elevated 2 h after injection only at ZT15. We identified significantly associated metabolites that correlated with exogenous 2-HB (Figures S7C and S7D; Data S7), including 106 liver and 127 muscle metabolites mostly involved in AA, sugar, and nucleotide metabolism.

To investigate a role for 2-HB in systemic metabolism, we administered unlabeled 2-HB to mice and monitored energy expenditure (EE) and respiratory exchange ratio (RER) by indirect calorimetry (Figure 7J). The higher dose (10 mmol/kg) acutely and transiently suppressed EE ~30 min after injection (Figure 7K). This effect was greater during the dark phase when circadian EE is at its highest (Dyar et al., 2018a). RER dropped acutely at night (Figure 7L) and remained low for several hours, suggesting an abrupt and persistent shift from glucose oxidation toward lipid utilization, as occurs during the light phase when mice are fasting. In support of our interpretation that 2-HB suppresses systemic glucose utilization, blood glucose was elevated 60 min after injection at ZT3 and ZT15 (Figure 7M). Thus, time- and exercise-dependent metabolites like 2-HB might communicate energy status under conditions of energy stress and impact tissue metabolism and systemic energy homeostasis.

## DISCUSSION

This resource adds spatiotemporal insights about the relative effects of exercise on tissue and systemic metabolism and temporal perspectives about the relative impact of performing exercise at different times of the day. The cross-tissue correlations highlight robust metabolic crosstalk between muscle and liver upon exercise in a time-dependent manner, with early active phase exercise at ZT15 having a greater impact on muscle-liver coordination. The liver plays an essential role as an energy producer during fasting and exercise, with major roles in glycogenolysis, gluconeogenesis, lipolysis, and ketogenesis (Rui, 2014; Warner et al., 2020). During early rest phase exercise at ZT3, when hepatic energy stores are enriched, the liver is primarily committed to energy metabolite production via glycogen and lipid degradation for skeletal muscle. Conversely, during early active phase exercise at ZT15, when hepatic glycogen content is reduced, skeletal muscle requires alternative energy sources. We observed reduced glucose in muscle and liver, lipolysis in WAT, and systemic activation of ketone body metabolism and AA breakdown upon exercise at ZT15. This local energy demand in skeletal muscle, coupled with temporal variations of hepatic glycogen storage and feeding-fasting cycles, appears to result in a tighter metabolic coordination between tissues. Future studies examining synergistic effects between nutritional state and exercise are warranted to ascertain which factors are more prominent for the time-of-day-dependent impact of exercise: fed/fasted cycles or circadian cycles.

Several metabolites act as stimuli for cellular energy sensors and signaling molecules, thus causing adaptive rewiring of signaling pathways and transcriptional networks (Dai et al., 2020). Metabolites and energy sensors can directly regulate circadian clock function (Nakahata et al., 2009; Ramsey et al., 2009). Activation of the energy sensor AMPK directly phosphorylates and destabilizes circadian transcriptional repressors CRY1/2, leading to circadian and gluconeogenic gene reprogramming (Lamia et al., 2009). Our study revealed a time- and muscle-specific impact of exercise on the accumulation of 5′-AMP, simultaneously leading to muscle-specific activation of AMPK. Additional exercise-stimulated molecules transduce metabolic information and mediate circadian reprogramming, including histone modifiers (SIRT1, HDACs) and transcription factors (HIF1α, GR, PPARs) (Adamovich et al., 2017; Guan et al., 2018; Peek et al., 2017; Quagliarini et al., 2019). Treadmill exercise in mice and electrical stimulation of cultured muscle cells can differentially advance or delay the circadian clock phase depending on the time of intervention (Kemler et al., 2020). Thus, exercise may reset misaligned muscle clocks if timed appropriately, for example, under fasted conditions. Our resource can be further mined for additional information about potential clock modifiers and can serve as a first step toward identifying the full complement of tissue and systemic metabolite messengers mediating clock entrainment.

By examining tissues in a physiological context, our multitissue metabolomics atlas can shed light on the time and tissue dependence of exerkines (Murphy et al., 2020). A limitation of our study is the timing of sample collection, as peak exerkine production and tissue distribution may be outside of the time intervals we explored. Future studies implementing different modes of exercise, higher temporal resolution, or additional arteriovenous sampling of exerkine-producing and target tissues will provide further physiological context. Nevertheless, we found exerkines present in different tissues in a time- and context-dependent manner. Additional work is needed to clarify the relative physiological importance of these exerkines, as well as tissue specificity, and how they communicate metabolic state between tissues. Our resource can serve as a starting point by indicating where exerkines may first arise and when their presence may be most physiologically relevant. As new metabolite exerkines are identified, our dataset can be revisited to test and explore possible interactions.

We identified 2-HB as an exercise-induced metabolite that may reflect the cytosolic redox state and energy stress at a systemic level. 2-HB levels were elevated in all tissues but only after early active phase exercise at ZT15. Selective accumulation of 2-HB indicates exercise at ZT15 evoked systemic metabolic stress. Normally considered a byproduct of GSH production or threonine catabolism, 2-HB also accumulates when mitochondrial metabolism of its precursor 2-KB is impaired (Miyazaki et al., 2015) or in response to an elevated NADH/NAD^+^ ratio due to increased lipid oxidation. In this context, 2-KB can serve as an electron acceptor, forming 2-HB and regenerating NAD^+^ through oxidation of cytosolic NADH by LDH (Gall et al., 2010; Landaas, 1975; Lord and Bralley, 2008; Miyazaki et al., 2015). 2-HB is a biomarker of cardiometabolic traits, mitochondrial dysfunction, lactic acidosis, ketoacidosis, prolonged fasting, and the maintenance of redox homeostasis and cell survival under stressed conditions (Gall et al., 2010; Goodman et al., 2020; Hsu et al., 2020; Liu et al., 2018; Thompson Legault et al., 2015). We previously identified 2-HB as a 24 h oscillating metabolite in skeletal muscle, liver, and serum (Dyar et al., 2018b). We report that circulating (exogenous) 2-HB transiently reduced EE and altered fuel use in a time-dependent manner. This resulted in increased glycemia and changes in liver and muscle metabolism. Thus, 2-HB may limit glucose utilization during energy stress and possibly clarify its strong association with type 2 diabetes risk.

In conclusion, this resource adds essential perspectives regarding the time-of-day-specific effects of exercise on systemic metabolism and tissue crosstalk. The production and consumption of skeletal muscle and liver metabolites during exercise changes according to the time of day, with 2-HB contributing as a systemic “exerkine” during exercise. This atlas will serve to deepen the understanding of the pleiotropic effects of exercise and uncover mechanistic insights to maximize the benefits of exercise on metabolic health.

### Limitations of study

In this study, we did not specifically consider the impact of sex, age, or metabolic disease in assessing the effects of exercise on systemic metabolism and tissue crosstalk. Our study was limited to a mouse model subjected to treadmill running, and results in humans, as well as the response to different exercise modalities, may vary. Although we assessed co-incident changes in metabolite levels in seven tissues and serum, as well as A/V differences in the liver and hindlimb muscle in response to acute exercise, future studies using tracer approaches will be useful to fully resolve the dynamic “metabolic circuitry” and “tissue crosstalk” in the whole organism.

## STAR★METHODS

### RESOURCE AVAILABILITY

#### Lead Contact

Further information and requests for resources and reagents should be directed to and will be fulfilled by the Lead Contact, Juleen R. Zierath (Juleen.Zierath@ki.se).

#### Materials Availability

This study did not generate new unique materials.

### EXPERIMENTAL MODEL AND SUBJECT DETAILS

Animal experiments complied with the European directive 2010/63/EU of the European Parliament and were approved by the Danish Animal Experiments Inspectorate (2012-15-2934-26 and 2015-15-0201-796). 2-HB experiments were approved by the local animal welfare authority in Germany (District government of upper Bavaria No.55.2-2532.Vet_2-17-125). For exercise experiments, specific Pathogen Free (SPF) male C57BL6/JBomTac mice were purchased from Taconic Biosciences and maintained at the animal facilities of the University of Copenhagen, Denmark. For 2-HB validation studies, SPF male C57BL6/J mice were purchased from Janvier and maintained at the animal facilities of the Helmholtz Zentrum Muünchen.

### METHOD DETAILS

#### Animals, Exercise, and Sample Collection

Mice were reared at 22±1°C with 12h light/dark cycle (light on at 6AM (ZT0) and light off at 6PM (ZT12)) and fed a standard rodent laboratory diet (#1310, Altromin, Germany) *ad libitum*. At 10-11 weeks of age, the mice were divided into two different groups of exercise or sham-exercise treatment either during the early rest phase group (ZT3) or the early active phase group (ZT15). Exercised mice in the early rest phase group were subjected to a single-bout of acute exercise for 1h at ZT3, whereas exercised mice in the early active phase group were subjected to the exercise for 1h at ZT15. Sedentary control mice for corresponding exercised mice at the different daily times were placed on a stationary treadmill for 1h (sham-exercise). The exercise protocol has been described previously (Brandauer et al., 2015; Sato et al., 2019; Treebak et al., 2014). Briefly, the mice were subjected to a single-bout of acute treadmill running (Columbus Instruments, OH) following a 4-day acclimatization protocol as described below;

*Day 1* (15 min total; 5% incline)
For the first 5 min, mice were placed on the stationary treadmill.
Start running at 6 m/min and accelerate by 2 m/min every 2 or 3 min up to 12 m/min
*Day 2* (15 min total; 5% incline)
Start running at 6m/min and accelerate by 2 m/min every 3 min up to 14 m/min
*Day 3* (15 min total; 5% incline)
Start running at 6m/min and accelerate by 2 m/min every 2 or 3 min up to 16 m/min
*Day 4*
Rest
*Day 5* (60 min total; 5% incline)
Start running at 6 m/min and accelerate by 2 m/min every 2 min up to 16 m/min
Electrical grids forced mice to keep running for the corresponding duration of exercise.

Under isoflurane anesthesia, skeletal muscle and liver were collected 0, 4, 8, 12, 16, and 20h after 1hr of exercise or sham-exercise performed during the early rest (ZT3) or active phase (ZT15). Serum, heart, hypothalamus (HT), epididymal white adipose tissue (eWAT), inguinal WAT (iWAT), and brown adipose tissue (BAT) were collected immediately after 1hr of exercise or sham-exercise performed during the early rest (ZT3) or active phase (ZT15). All tissues were immediately snap frozen in liquid nitrogen and stored at −80°C for subsequent use. For blood sampling across hindlimb skeletal muscles and liver, separate cohorts of 10-12-week-old SPF male C57B6/JBomTac mice were subjected to the same acute exercise or sham-exercise protocol either at ZT3 or ZT15 and then anesthetized.

##### Arteriovenous sampling across hindlimb skeletal muscles

Collection of venous blood from the skeletal muscle was drawn from the right common iliac vein using a 1 ml syringe and a 27 G needle. For arterial blood sampling the blood was drawn from the abdominal aorta using a catheter (BD Insyte Autoguard, 24 GA 0.75 IN, 0.7 x 19 mm, BD, Denmark). After the blood sampling, the gastrocnemius muscle was dissected and immediately snap frozen in liquid nitrogen before storage at −80°.

##### Blood sampling across the liver

The blood flow from the lower extremities and other organs that feed into the vena cava, apart from the liver, was occluded by a ligature placed around the vena cava inferior and aorta above the renal veins. Blood samples were then collected from the portal vein using a 1 ml syringe and 27 G needle, and blood from liver was drawn, after opening of the thorax, through a catheter similar to the catheter used for arterial blood sampling, inserted into the inferior caval vein, via the heart, and advanced to the liver. To avoid blood flow from the upper extremities a ligature was placed around the catheter in the vena cava. The liver was dissected immediately, and the samples were snap frozen in liquid nitrogen and stored at −80°C for subsequent use.

#### Global Metabolomics Profiling

Metabolomic analysis was performed by Metabolon (Durham, NC).

##### Sample Preparation

Samples were prepared using the automated MicroLab STAR system (Hamilton, NV). To remove protein, dissociate small molecules bound to protein or trapped in the precipitated protein matrix, and to remove chemically diverse metabolites, proteins were precipitated with methanol under vigorous shaking for 2 min (GenoGrinder, 2000, Glen Mills, NJ) followed by centrifugation. The resulting extract was divided into five fractions: two for analysis by two separate reverse phase (RP)/ultra-high-performance liquid chromatography-tandem mass spectrometry (UPLC-MS/MS) with positive ion mode electrospray ionization (ESI), one for analysis by RP/UPLC-MS/MS with negative ion mode ESL, one for analysis by HILIC/UPLC-MS/MS with negative ion mode ESI, and once sample was reserved for backup. Samples were placed briefly on a Zymark TurboVap (Caliper Life Sciences, CA) to remove the organic solvent.

##### UPLC-MS/MS

All methods utilized a Waters ACQUITY ultra-performance liquid chromatography and a Q-Exactive high resolution/accurate mass spectrometer (ThermoFisher Scientific) interfaced with a heated electrospray ionization (HESI-II) source and Orbitrap mass analyzer operated at 35,000 mass resolution. The sample extract was dried then reconstituted in solvents compatible to each of the four methods. Each reconstitution solvent contained a series of standards at fixed concentrations to ensure injection and chromatographic consistency. One aliquot was analyzed using acidic positive ion conditions, chromatographically optimized for more hydrophilic compounds. In this method, the extract was gradient eluted from a C18 column (Waters UPLC BEH C18-2.1x100 m, 1.7 μm) using water and methanol, containing 0.05% perfluoropentanoic acid (PFPA) and 0.1% formic acid. Another aliquot was also analyzed using acidic positive ion conditions, however it was chromatographically optimized for more hydrophobic compounds. In this method, the extract was gradient eluted from the same afore mentioned C18 column using methanol, acetonitrile, water, 0.05% PFPA and 0.01% formic acid and was operated at an overall higher organic content. Another aliquot was analyzed using basic negative ion optimized conditions using a separate dedicated C18 column. The basic extracts were gradient eluted from the column using methanol and water, however with 6.5 mM Ammonium Bicarbonate at pH 8. The fourth aliquot was analyzed via negative ionization following elution from a HILIC column (Waters UPLC BEH Amide 2.1 x 150 mm, 1.7 μm) using a gradient consisting of water and acetonitrile with 10 mM Ammonium Formate, pH 10.8.

##### Data Extraction, Compound Identification, Metabolite Quantification, and Data Normalization

Raw data were extracted, peak-identified and quality control processed using Metabolon’s proprietary hardware and software (Evans et al., 2009; Ford et al., 2020). Compounds were identified by comparison to library entries of purified standards or recurrent unknown entities. Biochemical identifications are based on three criteria: retention index within a narrow retention time/index window of the proposed identification, accurate mass match to the library +/− 10 ppm, and the MS/MS forward and reverse scores between the experimental data and authentic standards. The MS/MS scores are based on a comparison of the ions present in the experimental spectrum to the ions present in the library spectrum. Additional mass spectral entries have been created for structurally unnamed biochemicals, which have been identified by virtue of their recurrent nature. Peaks were quantified using area-under-the-curve. For measurements spanning multiple days, a data normalization step was performed to correct variation resulting from instrument inter-day tuning differences. All the samples in a particular tissue type were run in the same batch using the same instrument, so minimal instrument technical variation is present. Samples were run in a balanced manner using the group ID. Essentially, each compound was corrected in run-day blocks by registering the medians to equal one (1.00) and normalizing each data point proportionately. Following additional normalization (to mass extracted for BAT and hypothalamus, and to volume for serum), log transformation and imputation of missing values with the minimum observed value for each compound, Mixed Model Contrasts were used to identify biochemicals that differed significantly between groups. Imputation is applied within each sample type (muscle, liver, serum, etc.) regardless of the sampling timing and exercise condition. Imputation is applied to all the metabolites that are not detected (due to metabolites being under the threshold of detection (LOD)) in a per sample basis. If a metabolite is detected it is quantified (raw area counts) automatically. If there was only one value, it was still imputed with the minimum (this single value), but because the values would all be the same post-imputation, significance testing would not be performed on those metabolites (Missing values: 6.4% Muscle, 4.1% Serum, 4.0% Liver, 3.9% Heart, 5.0% HT, 13.1% eWAT, 5.8% iWAT, and 4.7% BAT).

##### Exclusion of metabolites related to xenobiotics

For differential analyses of metabolites identified and changed under experimental conditions (Figures 1B–1D, S1A–S1D, and 6D), all metabolites named and categorized according to Metabolon superpathways – amino acids, carbohydrates, energy, peptide, lipid, nucleotide, cofactors and vitamins, and xenobiotics –, and unnamed metabolites were included. For subsequent metabolite classification, pathway enrichment, and metabolite correlation analyses, metabolites categorized as xenobiotics and unnamed metabolites were excluded.

#### Targeted Gas Chromatography Mass Spectrometry (GC-MS)

Skeletal muscle (quadriceps) and liver samples collected after single-bout acute exercise or sham-exercise were used for further identification and quantification of polar metabolome biomarkers, including 2-hydroxybutyrate (2-HB) and 2-hydroxyisobutyrate (2-HIB), by targeted GC-MS. Preparation of liver and muscle tissue is based on prior methods, modified to suit the experimental design and analytical instruments (Luukkonen et al., 2017). Each tissue sample was transferred from −80°C into a Covaris tissueTUBE (520140), immersed in liquid nitrogen, and crushed with Covaris CryoPrep impactor CP02. Around 10 to 20 mg of powdered tissue were then transferred into 1.5 ml Eppendorf tubes and used for extraction. Exact mass was measured and written down for post-processing and normalization. Cryogenically frozen tissue powder was mixed with 60 μl of methanol containing internal standards: L-Valine-d8; 20 mg/l, Succinic acid-2,2,3,3-d4; 20 mg/l, L-Glutamic acid-^13^C_5_^15^N; 100 mg/l, D-3-hydroxybutyrate (^13^C_4_); 10 mg/l, Palmitic acid-d31; 100 mg/l. Additional 340 μl of methanol was added to the mixture. The suspension was vortexed and sonicated in ice-cold ultrasound bath for 15 min to facilitate metabolite extraction. After 15 min-incubation at 4°C for protein precipitation, the suspension was centrifuged for3 min on 10,000 rpm at 4°C and 200 μl of extract was aliquoted. Additional 50 ml was taken to create a pooled sample dedicated for quality control. Extract was dried under a stream of nitrogen with 6 l/min flow for 45 min. Native standards of 2-HB and 2-HIB were dissolved in methanol and diluted into calibration curve series, which was used for metabolite identification and quantification. Automatic derivatization was made using Gerstel MPS robot (Hartonen et al., 2013). Mox derivatizing agent (25 μl) was added to dried extract followed by 1h derivatization on 45°C. In a second step, 25 μl of BSTFA containing 1% TMCS was added and the same reaction conditions were used. Before the injection, 50 μl of hexane containing 10 mg/l of 4,4-dibromooctafluorobiphenyl was added. Compound was used to control the precision of 1 μl injection. Samples were analyzed using a gas chromatographer coupled with time of flight mass spectrometer (Leco Pegasus BT). Metabolites were separated on Restek Rxi 5ms column (pn:13423-6850) with Helium flow of 1.2 ml/min and 270°C inlet temperature. Temperature gradient started with 40°C where it was stable for a minute. After that, temperature was increased with a rate of 20°C/min until it reached 340°C, where it was stable for additional 3 min. Detector acquired mass spectra in scan mode, in the range from 50 to 750 Da with 10 Hz speed. To avoid solvent peak, acquisition started after 320s. MS was equipped with an EI source with standard 70eV fragmentation.

#### In Vivo Administration of 2-Hydroxybutyrate (2-HB)

All experiments were performed on 3-month-old male C57BL/6J mice from Janvier housed at room temperature (23°C) under a 12h light:12h dark cycle with standard chow diet (#1310, Altromin, Germany) and water provided ad libitum. Labeled 2-HB (2,3,3-d_3_), unlabeled 2-HB, or vehicle (saline) were administered by subcutaneous injection at the indicated doses and times. Tissues were collected at the indicated times immediately after cervical dislocation, snap frozen in liquid nitrogen and stored at −80°C for subsequent use. Serum was collected from trunk blood immediately chilled on ice, centrifuged at 1500 *g* and 4°C, and stored at −80°C. Serum glucose was determined by AU480 Chemistry Analyzer (Beckman Coulter). Energy expenditure and respiratory exchange ratio were assessed using a combined indirect calorimetry system (TSE Systems).

#### Matrix-Assisted Laser Desorption/Ionization Mass Spectrometry (MALDI-MS) Imaging

Frozen tissue samples were cryosectioned into 12 μm sections using Microm560 (Microm International, Walldorf, Germany) and thaw mounted onto indium tin oxide-coated conductive slides (Bruker Daltonics, Bremen, Germany). The slides were pre-treated with 1:1 poly-l-lysine (Sigma-Aldrich, Munich, Germany) and 0.1% Nonidet P-40 (Sigma) before mounting. Measurements were conducted, as described (Aichler et al., 2017; Kunzke et al., 2020). Briefly, the samples were covered with 10 mg/ml 1,5-diaminonaphthalene (DAN) matrix (Sigma-Aldrich) in 70% acetonitrile, using a SunCollect sprayer (Sunchrom, Friedrichsdorf, Germany). Data were acquired in negative ion mode using a Bruker Solarix 7.0 T Fourier-transform ion cyclotron resonance (FTICR) mass spectrometer (Bruker Daltonics) over a mass range of 50–650*m/z* and at a lateral resolution of 60 μm. SCiLS Lab (v. 2021b Pro) was used for peak picking and exported imzML files were processed with SPACiAL (Prade et al., 2020). After acquisition of the mass spectrometry data, the matrix was removed with 70% ethanol, and the tissue sections were stained with haematoxylin and eosin, coverslipped, scanned with a Mirax desk slide scanner (Zeiss, Göttingen, Germany) using a 20× magnification objective, and co-registered with the respective mass spectrometry imaging data. Metabolites were annotated with the Kyoto Encyclopedia of Genes and Genomes (KEGG) (Kanehisa and Goto, 2000) and Compass IsotopePattern (Bruker), allowing a mass error of 4 ppm, M-H adducts, M-H_2_O-H adducts, M+Na-2H adducts, M+K-2H adducts, and M+Cl adducts.

#### Real-Time Quantitative Polymerase Chain Reaction (Real-Time qPCR)

Using TRIzol reagent (ThermoFisher Scientific), total RNA was extracted from liver collected immediately after exercise or sham-exercise at each ZT3 and ZT15. One mg of total RNA was reverse transcribed to cDNA using Maxima H Minus Mastermix (ThermoFisher Scientific). cDNA was applied to real-time qPCR using PowerUp SYBR Master Mix (ThermoFisher Scientific). Gene expression was normalized to 18S rRNA. Primers used for qPCR are listed below;

m*Gys2* For 5’-ccagacaaattccacctagagc-3’, Rev 5’-gggcctgggatacttaaagc-3’
m*Pygl* For 5’-ccagagtgctctaccccaat-3’, Rev 5’-ccaccacaaagtactcctgtttc-3’
m*G6pc* For 5’-tctgtcccggatctaccttg-3’, Rev 5’-gaaagtttcagccacagcaa-3’
m*Pck1* For 5’-tgttggctggctctcactgac-3’, Rev 5’-gggaacctggcgttgaatgc-3’
m*Hk2* For 5’-caactccggatgggacag-3’, Rev 5’-cacacggaagttggttcctc-3’
m*Pdk4* For 5’-gagctggtatatccagagcctgat-3’, Rev 5’-cgaactttgaccagcgtgtct-3’
m*Ppara* For 5’-acaaggcctcagggtacca-3’, Rev 5’-gccgaaagaagcccttacag-3’
m*Ppard* For 5’-gtatgcgcatgggactcac-3’, Rev 5’-gtctgagcgcagatggact-3’
m*Acss1* For 5’-ccaccaagatcgccaagta-3’, Rev 5’-atctggttttggggagacg-3’
m*Slc16a1* For 5’-ggatatcatctataatgttggctgtc-3’, Rev 5’-gctgccgtatttattcaccaa-3’
m*Pnpla2* For 5’-gtgcatatgccctttcctgt-3’, Rev 5’-ttctgatctctcccaaggttct-3’
m*Plin4* For 5’-ggacttacaaacagcaacagacc-3’, Rev 5’-tctgtgagttggtggacacttt-3’
*18S rRNA* For 5’-cgccgctagaggtgaaattc-3’, Rev 5’-cgaacctccgactttcgttct-3’.

#### Western Blot

Whole cell lysates were extracted from skeletal muscle, liver, and heart using the buffer containing 137 mM NaCl, 2.7 mM KCl, 1 mM MgCl_2_, 5 mM Na_4_P_2_O_7_, 10mM NaF, 1% Triton X-100, 10% glycerol, 20 mM Tris pH7.8, 1 mM EDTA, 0.2 mM PMSF, 0.5 mM Na_3_VO_4_, 10 mM NAM, 0.33 μM TSA, and 1X protein inhibitor cocktail (Roche). Whole cell lysates were extracted from eWAT as described (Diaz Marin et al., 2019). Twenty μg of lysate per sample was applied to SDS-PAGE. Anti-phospho AMPK*α* (Cell Signaling Technology, 2535), anti-AMPK*α* (Cell Signaling Technology, 2603), and anti-p84 (Genetex, GTX70220) antibodies were used for Western blot analyses.

#### Myotube Contraction and Targeted Liquid Chromatography Mass Spectrometry (LC-MS)

C2C12 myoblasts (#CRL-1772; American Type Culture Collection) were seeded into 35 mm at a density of 10^5^ cells/well, cultured in Dulbecco’s Modified Eagle’s Medium (DMEM, 25 mM glucose) supplemented with 10% fetal bovine serum (FBS), and 1% penicillin-streptomycin at 37°C, 5% CO_2_. After reaching ~90% confluence, differentiation was induced by switching from 10% FBS to 2% horse serum. Differentiation medium was changed every 2nd day until fully differentiated myotubes could be visualized under a light microscope (5–6 days). On day 6 of differentiation, cells were washed in warm PBS and the media was changed to a standard Krebs Ringer Phosphate buffer supplemented with 0.2% BSA and 20 mM glucose or20 mM mannitol (used to prevent osmotic stress) for a total of 6h. Electrical pulse stimulation (EPS) was applied to the cells in culture to induce contraction in the last 3 hours of incubation using the Ion-optics system C-dish electrode in a six-well format. The EPS protocol consisted of 3h with 1 Hz frequency, 2 ms in duration and 30 V. Immediately after the end of the contraction protocol the cells were transferred to ice, washed with cold PBS and metabolites were extracted and the cells scraped in 150 μl of ice-cold 0.5 M perchloric acid. Samples were vortexed for 20s and then centrifuged at 14,000 *g* for 3 min at 4°C and then 100 μl of supernatant was neutralized with the addition of 25 μl of 2.3M KHCO_3_, centrifuged again at 14,000 *g* for 3 min at 4°C, and then 25 μl of the supernatant was transferred to an HPLC tube containing 25 μl of HPLC-grade methanol. LC-MS was performed on an ultra-performance liquid chromatography-tandem mass spectrometer (UPLC-MS-MS, ACQUITY UPLC coupled with Xevo TQ-XS, Waters Corporation, Milford, Massachusetts). The auto-sampler was set at 6°C and the column ACQUITY premier BEH Amide 1.7 μm VanGuard FIT 2.1 x 150 mm (Waters Corporation, Milford) temperature was maintained at 40°C. The total run time was 19 min at a flow rate of 200 μL/min. Initially, the gradient started with a 1.5 min at 90% mobile phase B (Acetonenitrile/H_2_O, 90/10 (v/v), 10 mM ammonium acetate, pH at 9), and then rising to 50% mobile phase A (H_2_O, 10 mM ammonium acetate, pH at 9) over the next 9.5 min. Mobile phase A was quickly increased to 65% in 0.5 min and maintained for 4 min and finally equilibrated to the initial conditions for 4 min. Both mobile phases were spiked with 5 μM methylenediphosphonic acid as a deactivator additive. An injection volume of 1 μL of sample extract was used. MRM transitions 268.1 > 136 and 348 > 136 were used for quantification for Adenosine and AMP respectively. Calibration curves of Adenosine (0.05 nM to 1 μM) and AMP (0.5 nM to 10 mM) were carried out for quantification. Limits of determination (LOD) were 5 nM for both compounds, defined by signal to noise ratio (S/N) > 3. The relative standard deviation (RSD) for both compounds was below 10 %. C2C12 experiments were performed twice on separate days and n number indicates individual wells.

### QUANTIFICATION AND STATISTICAL ANALYSIS

#### Statistical Analysis

For the statistical analysis of metabolite levels among different experimental conditions, two-way ANOVA (serum, heart, hypothalamus, eWAT, iWAT, and BAT) and three-way ANOVA (muscle and liver) Model Contrasts were used to identify biochemicals that differed significantly between experimental groups, following normalization, log transformation and imputation of missing values, if any, with the minimum observed value for each compound. Differences between two groups were considered statistically significant for p < 0.05. Data are presented as mean ± SEM. Storey’s q value method for estimating the False Discovery Rate (FDR) was used to correct for multiple testing. For the statistical analyses of gene expression and glycogen content, data are presented as mean ± SEM and were analyzed by Two-way ANOVA with Sidak’s post hoc testing where applicable (Prism 7.0). For the analysis of rhythmic metabolites in liver, the nonparametric test Bio_Cycle was used incorporating a window of 20-28 h for the determination of circadian periodicity (Agostinelli et al., 2016), including amplitude and phase analysis. A gene or metabolite was considered circadian based on a p value cutoff of 0.05. For single group comparisons, an unpaired two-tailed t test with a p value cutoff of 0.05 was used.

#### MetaboAnalyst

Metabolites positively or negatively associated with 2-HB were determined according to Spearman correlation adjusted p < 0.05 after multiple testing by Benjamini-Hochberg. Enrichment analysis of 2-HB associated metabolites was performed using MetaboAnalyst (Chong et al., 2019) with default parameters and Kyoto Encyclopedia of Genes and Genomes (KEGG) IDs. Quantitative Enrichment Analysis was performed via the Enrichment Analysis module using the pathway based KEGG metabolite set library (84 metabolite sets based on human metabolic pathways) or the main-class chemical structures library (464 main chemical class metabolite sets).

#### Metabolite Correlation and Network Statistics

Metabolite data were auto scaled before correlation analysis. Pearson’s correlation coefficient was calculated on log normalized data to estimate inter- and intra-tissue metabolite pair correlations. Networks are based on metabolite-pair correlations with an estimated p value < 0.0001. Undirected networks were analyzed and visualized using the Compound Spring Embedder (CoSE) layout of Cytoscape (Dogrusoz et al., 2009). Node degree refers to the number of connected neighbor nodes/metabolites. Node closeness was calculated using MATLAB 2020a centrality function as the reciprocal of the sum of the length of the shortest paths between the node and all other nodes in the graph.

#### Arterial and Venous (A/V) Differences

Peak area data were batch normalized. Serum data were normalized to volume extracted. Metabolite values greater than 4 times the standard deviation were considered as outliers and removed. Metabolites with more that 50% missing data were removed from the data. Data were log normalized and missing values were imputed using k-nearest-neighbor. Samples were auto-scaled. Significant tissue uptake or release was estimated using a paired t test for serum A/V data. Log-Fold change of tissue uptake/release was calculated based on replicate median values.

#### KEGG Enrichment Analysis

Metabolites for enrichment were selected based on ANOVA test for differences between groups (exercise vs sedentary, tissue profiling) or paired t test for significant uptake/release (A/V profiling), p values < 0.05. Metabolites were annotated based on Metabolon KEGG annotation. KEGG pathway definitions were downloaded from https://www.genome.jp, release 98.0. KEGG pathway significance was estimated using a hypergeometrical distribution test.

#### Data Processing and Quantification for Targeted GC-MS

Raw files were converted into centroid mode and exported as netCDF files. Metabolites without available standard were identified based on the spectral similarity and retention index criteria using in-house and NIST 17 spectral libraries. 0.85 of similarity score and less than 30 RI difference was used for successful annotation. Sugars with multiple hits were assigned to their structural group. Using spectral similarity of injected standards and putatively identified metabolites, we determined characteristic quantifier ions and retention time. Characteristic peaks were extracted and integrated using in-house script developed by Swedish Metabolome Center. Extraction was performed in targeted manner. Metabolites with calibration curves were normalized with group specific internal standards: L-Valine-d8, Succinic acid-2,2,3,3-d4, L-Glutamic acid-^13^C_5_^15^N, D-3-hydroxybutyrate (^13^C_4_) and Palmitic acid-d31, and compared with dilution series of native standards. Putatively identified metabolites were normalized to injection standard: 4,4-Dibromooctafluorobiphenyl. At the end, concentrations and peak areas were normalized to the initial amount of tissue. Results were expressed as μmol of compound in g of tissue for calibration curve covered metabolites and as normalized areas for putatively identified metabolites.

## Supplementary Material

Fig_S1-S7

Data_S1

Data_S2

Data_S3

Data_S4

Data_S5

Data_S6

Data_S7

Supplemental information can be found online at https://doi.org/10.1016/j.cmet.2021.12.016.

## Figures and Tables

**Table:** KEY RESOURCES TABLE

REAGENT or RESOURCE	SOURCE	IDENTIFIER
Antibodies		
Anti-phospho-AMPKα	Cell Signaling Technology	Cat# 2535; RRID: AB_331250
Anti-AMPKα	Cell Signaling Technology	Cat# 2603; RRID: AB_490795
Anti-p84	Genetex	Cat# GTX70220; RRID: AB_372637
Anti-Mouse IgG, HRP conjugate	EMD Millipore	Cat# AP160P; RRID: AB_92531
Anti-Rabbit IgG, HRP-linked	Cell Signaling Technology	Cat# 7074; RRID: AB_2099233
Chemicals, peptides, and recombinant proteins		
Methanol; LC-MS Ultra CHROMASOLV	Honeywell	14262-2L
Hexane; GC-MS SupraSolv	Supelco	1007951000
D-3-Hydroxybutyrate; Sodium (^13^C_4_)	Cambridge Isotope	CLM-3853-PK
L-Valine-d8	Sigma-Aldrich	486027-1G
Palmitic acid-d31	Cayman chemicals	1-800-364-9897
Succinic acid-2,2,3,3-d4	Sigma-Aldrich	293075-1G
L-Glutamic acid-^13^C_5_^15^N	Sigma-Aldrich	607851-250MG
4,4-Dibromooctafluorobiphenyl	Sigma-Aldrich	101990-1G
2-Hydroxybutyric acid; Sodium	Sigma-Aldrich	220116-5G
2-Hydroxyisobutyric acid	Sigma-Aldrich	323594-25G
3-Hydroxybutyric acid	Sigma-Aldrich	166898-1G
3-Hydroxyisobutyric acid; Sodium	Sigma-Aldrich	11161-100MG
L-Leucine	Sigma-Aldrich	AAS18-5ML
L-Isoleucine	Sigma-Aldrich	AAS18-5ML
L-Valine	Sigma-Aldrich	AAS18-5ML
L-Glutamic acid	Sigma-Aldrich	AAS18-5ML
L-Aspartic acid	Sigma-Aldrich	AAS18-5ML
L-Proline	Sigma-Aldrich	AAS18-5ML
L-Alanine	Sigma-Aldrich	AAS18-5ML
Oleic acid	Cayman Chemicals	90260
Stearic acid	Cayman Chemicals	10011298
Malic acid	Sigma-Aldrich	240176-50G
Lactic acid	Sigma-Aldrich	L6402-1G
Methoxamine (MOX)	Thermo Scientific	TS-45950
N,O-Bis(trimethylsilyl)trifluoroacetamide with 1% Trimethylchlorosilane (BSTFA + 1%TMCS)	Thermo Scientific	TS-38831
(+/−)-2-Hydroxybutyric acid-2,3,3-d3; Sodium	CDN isotopes	D-7002(lotAB-224)
Indium tin oxide-coated conductive slides	Bruker	8237001
Poly-l-lysine	Sigma-Aldrich	P8920-100ML
Nonidet P-40	Sigma-Aldrich	21-3277
1,5-diaminonaphthalene (DAN)	Sigma-Aldrich	56451-250MG
Hematoxylin	Leica	3801698A
Eosin	Leica	3801698D
Acetonitrile	Sigma-Aldrich	34851-2.5L
TRIzol	ThermoFisher Scientific	Cat# 15596026
Maxima H Minus Mastermix	ThermoFisher Scientific	Cat# M1662
PowerUp SYBR Master Mix	ThermoFisher Scientific	Cat# A25918
Protein Assay Dye Reagent	Bio-Rad Laboratories	Cat# 500-0006
Nitrocellulose Membrane, 0.45 μm	Bio-Rad Laboratories	Cat# 1620115
Immobilon Western Chemiluminescent HRP substrate	EMD Millipore	Cat# WBKLS0500
cOmplete, EDTA-free Protease Inhibitor Cocktail	Roche	Cat# 11873580001
Perchloric acid	Sigma-Aldrich	244252-100ML
D-Glucose	Sigma-Aldrich	G8270-1KG
D-Mannitol	Sigma-Aldrich	M4125-500G
DMEM, high glucose	ThermoFisher Scientific	11965084
Penicillin-Streptomycin	ThermoFisher Scientific	15140122
Horse Serum	Sigma-Aldrich	H1270-1L
Fetal Bovine Serum	ThermoFisher Scientific	16000
Deposited data		
Mouse skeletal muscle, sedentary, ZT3	http://circadiomics.igb.uci.edu/	MOUSE EXERCISE METABOLOME MORNING-SEDENTARY
Mouse skeletal muscle, exercise, ZT3	http://circadiomics.igb.uci.edu/	MOUSE EXERCISE METABOLOME MORNING-EXERCISE
Mouse skeletal muscle, sedentary, ZT15	http://circadiomics.igb.uci.edu/	MOUSE EXERCISE METABOLOME EVENING-SEDENTARY
Mouse skeletal muscle, exercise, ZT15	http://circadiomics.igb.uci.edu/	MOUSE EXERCISE METABOLOME EVENING-EXERCISE
Mouse liver, sedentary, ZT3	http://circadiomics.igb.uci.edu/	MOUSE HEPATIC MORNING-SEDENTARY
Mouse liver, exercise, ZT3	http://circadiomics.igb.uci.edu/	MOUSE HEPATIC MORNING-EXERCISE
Mouse liver, sedentary, ZT15	http://circadiomics.igb.uci.edu/	MOUSE HEPATIC EVENING-SEDENTARY
Mouse liver, exercise, ZT15	http://circadiomics.igb.uci.edu/	MOUSE HEPATIC EVENING-EXERCISE
Mouse muscle 24-hr metabolome, sedentary & exercise at ZT3 & ZT15	https://doi.org/10.17632/6x5vd4d5rd.1	24h_LiverMuscle
Mouse liver 24-hr metabolome, sedentary & exercise at ZT3 & ZT15	https://doi.org/10.17632/6x5vd4d5rd.1	24h_LiverMuscle
Mouse serum metabolome, sedentary & exercise, ZT3 & ZT15	https://doi.org/10.17632/6x5vd4d5rd.1	TissueProfiling
Mouse heart metabolome, sedentary & exercise, ZT3 & ZT15	https://doi.org/10.17632/6x5vd4d5rd.1	TissueProfiling
Mouse hypothalamus metabolome, sedentary & exercise, ZT3 & ZT15	https://doi.org/10.17632/6x5vd4d5rd.1	TissueProfiling
Mouse epididymal & inguinal white adipose tissue metabolomes, sedentary & exercise, ZT3 & ZT15	https://doi.org/10.17632/6x5vd4d5rd.1	TissueProfiling
Mouse brown adipose tissue metabolome, sedentary & exercise, ZT3 & ZT15	https://doi.org/10.17632/6x5vd4d5rd.1	TissueProfiling
A/V metabolome, skeletal muscle, sedentary & exercise, ZT3 & ZT15	https://doi.org/10.17632/6x5vd4d5rd.1	AV_LiverMuscle
A/V metabolome, hindlimb arterial blood, sedentary & exercise, ZT3 & ZT15	https://doi.org/10.17632/6x5vd4d5rd.1	AV_LiverMuscle
A/V metabolome, hindlimb venous blood, sedentary & exercise, ZT3 & ZT15	https://doi.org/10.17632/6x5vd4d5rd.1	AV_LiverMuscle
A/V metabolome, liver, sedentary & exercise, ZT3 & ZT15	https://doi.org/10.17632/6x5vd4d5rd.1	AV_LiverMuscle
A/V metabolome, portal vein, sedentary & exercise, ZT3 & ZT15	https://doi.org/10.17632/6x5vd4d5rd.1	AV_LiverMuscle
A/V metabolome, vena cava, sedentary & exercise, ZT3 & ZT15	https://doi.org/10.17632/6x5vd4d5rd.1	AV_LiverMuscle
Original Excel data sheets + WB pictures for figures	N/A	This paper (Data S1)
Transcriptomics data	(Sato et al., 2019)	GEO: GSE126962
Experimental models: Organisms/strains		
C57BL6/JBomTac mice	Taconic Biosciences	N/A
C57BL6/J mice (2-HB validation studies)	Janvier	N/A
C2C12 myoblasts	American Type Culture Collection	#CRL-1772
Oligonucleotides		
See Real-time qPCR section for qPCR primers	N/A	N/A
Software and Algorithms		
Prism 7.0	GraphPad Software	http://www.graphpad.com
Bio_Cycle	(Agostinelli et al., 2016)	http://circadiomics.igb.uci.edu/biocycle
CircadiOmics	(Ceglia et al., 2018; Patel et al., 2012)	http://circadiomics.igb.uci.edu
MATLAB and Statistics Toolbox R2020a	Mathworks	https://www.mathworks.com/products/new_products/previous_release_overview.html
MetaboAnalyst	(Chong et al., 2019)	https://www.metaboanalyst.ca/
Cytoscape	(Dogrusoz et al., 2009)	https://cytoscape.org/
Other		
Animal treadmill	Columbus Instruments	Exer 3/6
a standard rodent laboratory diet	Altromin	Cat# 1310
C-dish electrode system	Ion-optics	6-well

## Data Availability

24h Metabolomics data from skeletal muscle and liver are available from CircadiOmics (Ceglia et al., 2018; Patel et al., 2012). Excel datasheets used to create all graphs in the paper and uncropped scans of all the Western blot images presented in the paper are found in Data S1. Original and scaled metabolomics data can be found in Data S2. Raw and processed metabolomics data have also been deposited into Mendeley Data (https://doi.org/10.17632/6x5vd4d5rd.1).

## Highlights

- Time of exercise defines system-wide activation of energy metabolism
- Exercise timing rewires intra-tissue and inter-tissue metabolite correlations
- Maintenance of inter-tissue metabostasis is specified by exercise time
- Comparative analyses reveal time- and tissue-dependent exerkines
