# Supplementary material for: Atlas of exercise metabolism reveals time-dependent signatures of metabolic homeostasis
Source: Cell Metab. Author manuscript; Available in PMC 2026 May 20. (PMC13189211; doi:10.1016/j.cmet.2021.12.016)
Supplement: Fig_S1-S7 [file NIHMS2168542-supplement-Fig_S1-S7.pdf]

Cell Metabolism, Volume *34*

Supplemental information

Atlas of exercise metabolism reveals time-dependent signatures of metabolic homeostasis

Shogo Sato, Kenneth A. Dyar, Jonas T. Treebak, Sara L. Jepsen, Amy M. Ehrlich, Stephen P. Ashcroft, Kajetan Trost, Thomas Kunzke, Verena M. Prade, Lewin Small, Astrid Linde Basse, Milena Schönke, Siwei Chen, Muntaha Samad, Pierre Baldi, Romain Barrès, Axel Walch, Thomas Moritz, Jens J. Holst, Dominik Lutter, Juleen R. Zierath, and Paolo Sassone-Corsi

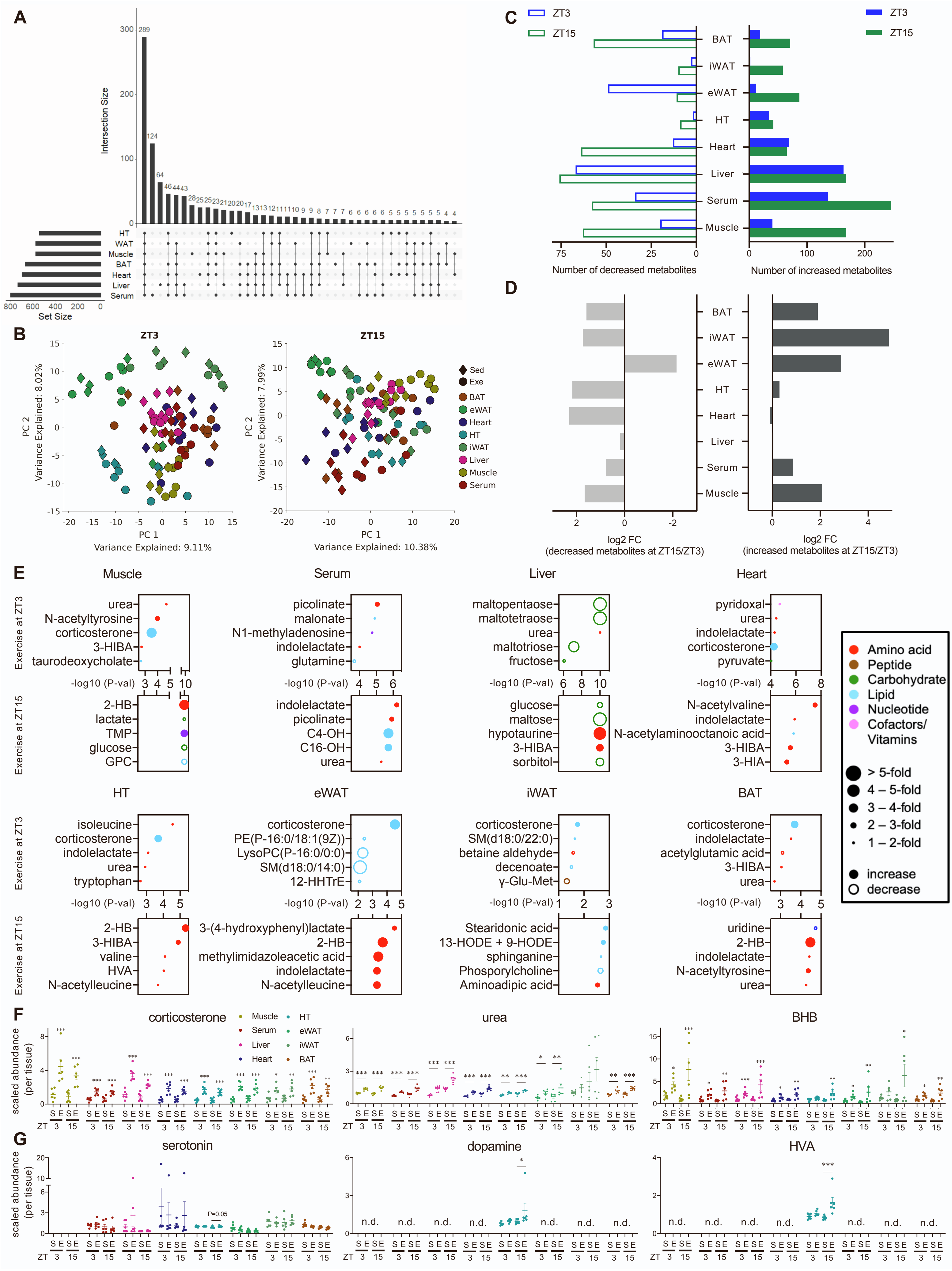
A
Intersection Size
289
124
64
46 44 43
28 25 25 23 21 20 20 17 13 13 12 11 11 10 9 9 8 7 7 6 6 6 6 5 5 5 5 5 5 5 4 4
HT
WAT
Muscle
BAT
Heart
Liver
Serum
Set Size
B
ZT3
ZT15
PC 2
Variance Explained: 8.02%
Variance Explained: 7.99%
PC 1
Variance Explained: 9.11%
Variance Explained: 10.38%
Sed
Exe
BAT
eWAT
Heart
HT
iWAT
Liver
Muscle
Serum
C
ZT3
ZT15
BAT
iWAT
eWAT
HT
Heart
Liver
Serum
Muscle
Number of decreased metabolites
Number of increased metabolites
D
log2 FC
(decreased metabolites at ZT15/ZT3)
log2 FC
(increased metabolites at ZT15/ZT3)
E
Muscle
Serum
Liver
Heart
Exercise at ZT3
urea
N-acetyltyrosine
corticosterone
3-HIBA
taurodeoxycholate
picolinate
malonate
N1-methyladenosine
indolelactate
glutamine
maltopentaose
maltotetraose
urea
maltotriose
fructose
pyridoxal
urea
indolelactate
corticosterone
pyruvate
-log10 (P-val)
Exercise at ZT15
2-HB
lactate
TMP
glucose
GPC
indolelactate
picolinate
C4-OH
C16-OH
urea
glucose
maltose
hypotaurine
3-HIBA
sorbitol
N-acetylvaline
indolelactate
N-acetylaminooctanoic acid
3-HIBA
3-HIA
HT
eWAT
iWAT
BAT
Exercise at ZT3
isoleucine
corticosterone
indolelactate
urea
tryptophan
corticosterone
PE(P-16:0/18:1(9Z))
LysoPC(P-16:0/0:0)
SM(d18:0/14:0)
12-HHTrE
corticosterone
SM(d18:0/22:0)
betaine aldehyde
decenoate
γ-Glu-Met
corticosterone
indolelactate
acetylglutamic acid
3-HIBA
urea
-log10 (P-val)
Exercise at ZT15
2-HB
3-HIBA
valine
HVA
N-acetylleucine
3-(4-hydroxyphenyl)lactate
2-HB
methylimidazoleacetic acid
indolelactate
N-acetylleucine
Stearidonic acid
13-HODE + 9-HODE
sphinganine
Phosporylcholine
Aminoadipic acid
uridine
2-HB
indolelactate
N-acetyltyrosine
urea
Amino acid
Peptide
Carbohydrate
Lipid
Nucleotide
Cofactors/
Vitamins
> 5-fold
4 – 5-fold
3 – 4-fold
2 – 3-fold
1 – 2-fold
increase
decrease
F
corticosterone
urea
BHB
Muscle
Serum
Liver
Heart
HT
eWAT
iWAT
BAT
scaled abundance
(per tissue)
S E S E
ZT 3 15
G
serotonin
dopamine
HVA
P=0.05
n.d.

Figure S1 Atlas of metabolome in response to exercise at different times of the day (related to Figure 1)
(A) Significantly altered metabolites in a specific tissue alone or in combination among different tissues. Vertical bars indicate set size, horizonal bars indicate intersection size, and dots and lines indicate set identity.
(B) PCA plots based on 289 metabolites common to all 7 tissues and serum. PCA plots of all metabolomics samples combined (only common metabolites were included) samples. Color of the dot refers to tissue and shape refers to sedentary or exercise condition.
(C) Bar charts show number of metabolites significantly decreased (right) and increased (left) upon exercise at ZT3 versus ZT15 in 7 tissues and serum.
(D) Relative decreased (left) and increased (right) metabolites after exercise at ZT15 to those after exercise at ZT3 in 7 tissues and serum.
(E) Top 5 metabolites in response to exercise at ZT3 versus ZT15.
Metabolomics data contain 5-6 individual tissues per group. Two-way ANOVA was applied for the detection of significantly altered metabolites using p-value cutoff of 0.05.
(F) Corticosterone, urea, beta-hydroxybutyrate (BHB) as time- and tissue-independent metabolites in response to exercise.
(G) Time-dependent impact of exercise on hypothalamic neurotransmitters serotonin, dopamine, and homovanillate (HVA). Data are represented as mean±SEM (n=5-6) and were analyzed by two-way ANOVA based on statistical methods of Metabolon (Sedentary versus Exercise at corresponding time of treatment). *$p<0.05$, **$p<0.01$, and ***$p<0.001$. S; Sedentary, E; Exercise.

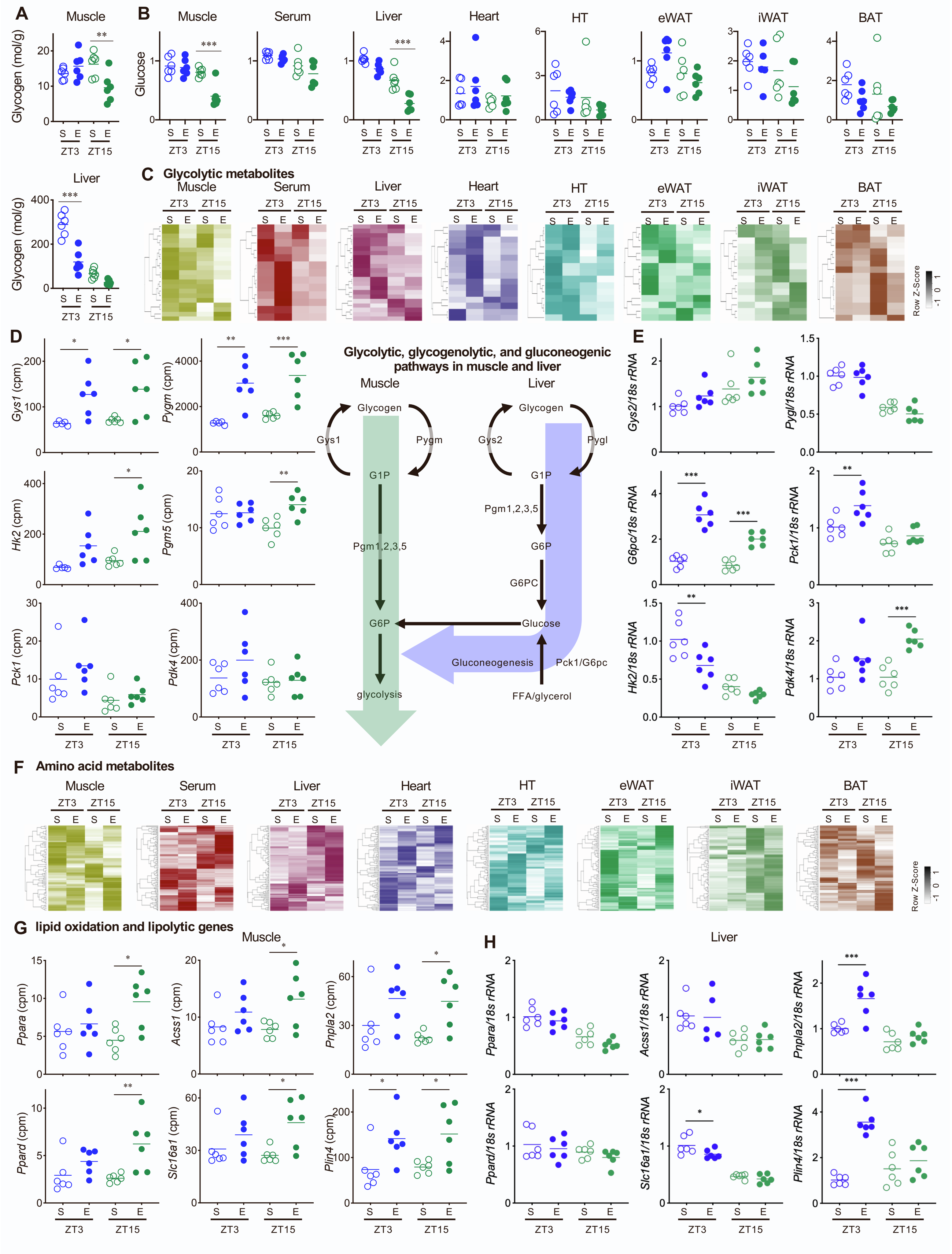
A
Muscle
Glycogen (mol/g)
Liver
B
Muscle
Serum
Liver
Heart
HT
eWAT
iWAT
BAT
Glucose
S E S E
ZT3 ZT15
C Glycolytic metabolites
Row Z-Score
D
Gys1 (cpm)
Pygm (cpm)
Hk2 (cpm)
Pgm5 (cpm)
Pck1 (cpm)
Pdk4 (cpm)
Glycolytic, glycogenolytic, and gluconeogenic pathways in muscle and liver
Muscle
Liver
Glycogen
Gys1
Pygm
Gys2
Pygl
G1P
Pgm1,2,3,5
G6P
G6PC
Glucose
Gluconeogenesis
Pck1/G6pc
FFA/glycerol
glycolysis
E
Gys2/18s rRNA
Pygl/18s rRNA
G6pc/18s rRNA
Pck1/18s rRNA
Hk2/18s rRNA
Pdk4/18s rRNA
F Amino acid metabolites
G lipid oxidation and lipolytic genes
Muscle
Ppara (cpm)
Acss1 (cpm)
Pnpla2 (cpm)
Ppard (cpm)
Slc16a1 (cpm)
Plin4 (cpm)
H
Liver
Ppara/18s rRNA
Acss1/18s rRNA
Pnpla2/18s rRNA
Ppard/18s rRNA
Slc16a1/18s rRNA
Plin4/18s rRNA

Figure S2 Spatiotemporal impacts of exercise on metabolism (Related to Figure 2)
(A) Glycogen amount in skeletal muscle and liver isolated mice after exercise at ZT3 versus ZT15 (Sato et al., 2019).
(B) Glucose levels are detected by MS in skeletal muscle, serum, liver, heart, HT, eWAT, iWAT and BAT from mice subjected to exercise at ZT3 versus ZT15.
(C) Heatmaps displaying metabolites related to glycolysis and TCA cycle in skeletal muscle, serum, liver, heart, HT, eWAT, iWAT and BAT.
(D) Glycogenolytic, glycolytic and gluconeogenic gene expression in skeletal muscle determined using published transcriptomic data (GSE126962) (Sato et al., 2019).
(E) Glycogenolytic, glycolytic and gluconeogenic gene expression in liver determined by qPCR.
(F) Heatmaps displaying metabolites related to amino acid metabolism in skeletal muscle, serum, liver, heart, HT, eWAT, iWAT, and BAT.
(G and H) Gene expression related to lipid metabolism upon exercise at ZT3 and ZT15 in skeletal muscle and liver.
(G) Selective activation of lipid oxidation pathway in skeletal muscle after exercise at the early active phase versus the early rest phase as revealed by our published (Sato et al., 2019) skeletal muscle transcriptome after exercise at ZT3 and ZT15 (GSE126962).
(H) Selective activation of lipolytic pathway in liver after exercise at the early rest phase versus the early active phase revealed by qPCR.
Metabolomics data contain 5-6 individual tissues per group. Two-way ANOVA was applied for the detection of significantly altered metabolites using p-value cutoff of 0.05. Data are represented as mean±SEM (n=6) and were analyzed by two-way ANOVA using Bonferroni *post hoc* testing (Sedentary versus Exercise at corresponding time of treatment). *$p<0.05$, **$p<0.01$, and ***$p<0.001$. S; Sedentary, E; Exercise.

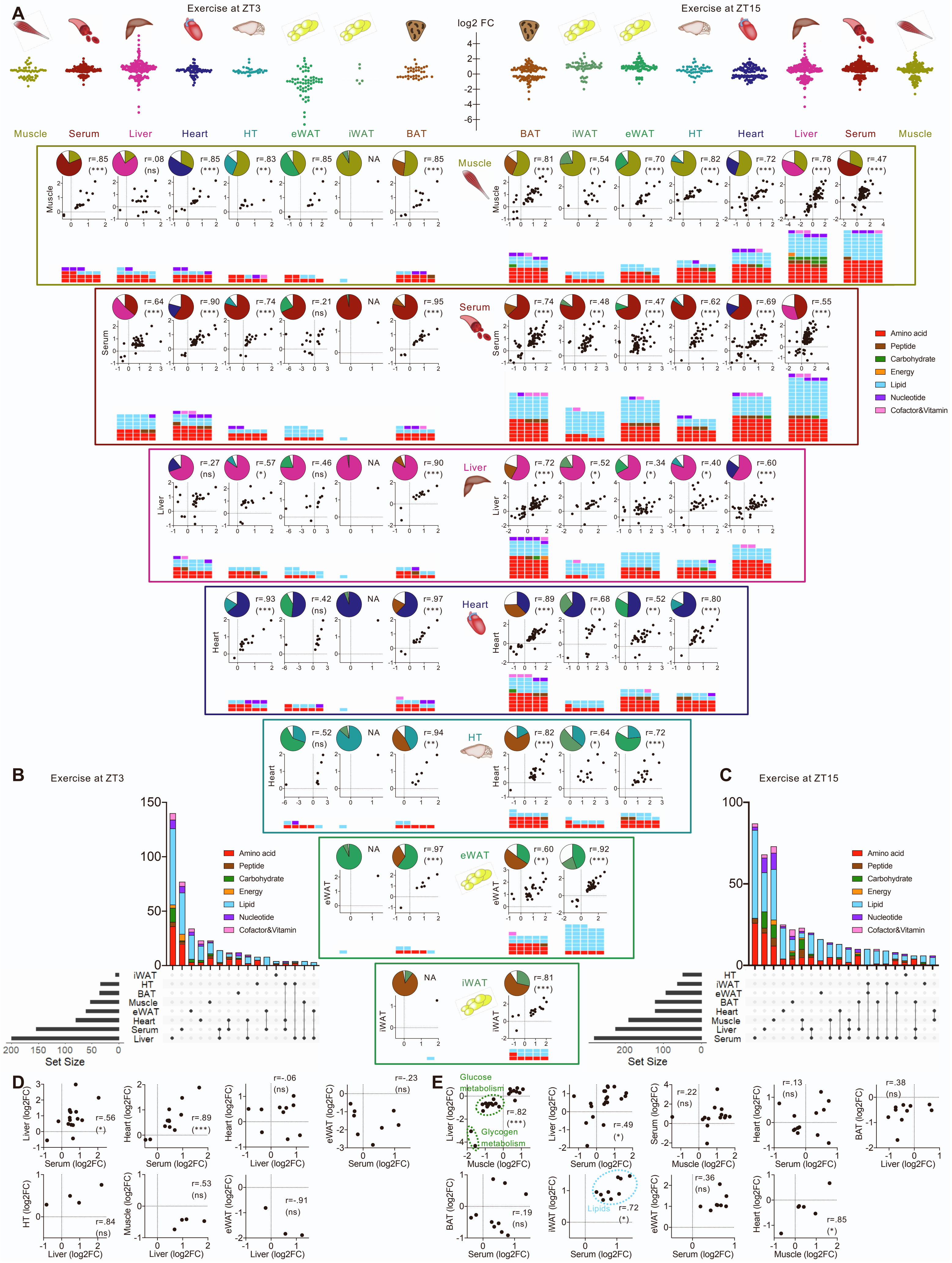

A
Exercise at ZT3
Exercise at ZT15
log2 FC
Muscle
Serum
Liver
Heart
HT
eWAT
iWAT
BAT
Amino acid
Peptide
Carbohydrate
Energy
Lipid
Nucleotide
Cofactor&Vitamin
B
Exercise at ZT3
Set Size
C
Exercise at ZT15
D
E
Glucose metabolism
Glycogen metabolism
Lipids

Figure S3 Integration and comparison of time of day-dependent effects of exercise across different tissues (Related to Figures 1 and 2)

(A) Tissue-tissue correlation of metabolome in response to exercise at ZT3 (left) and ZT15 (right). Upper chart, each dot is a metabolite significantly increased or decreased by exercise at ZT3 (left) and ZT15 (right). Pie charts, comparing the ratio of common (white pieces) and tissue-specific metabolites altered after exercise between two tissues. Correlation charts, displaying metabolites changed commonly in the combination of each tissue after exercise at ZT3 (left) and ZT15 (right). Correlation coefficient and significance are measured based on the Pearson correlation coefficient. *$p<0.033$, **$p<0.002$, and ***$p<0.001$. Waffle plots, showing biological classification of correlated metabolites changed after exercise at ZT3 (left) and ZT15 (right) between two tissues. Each square indicates each metabolite.

(B and C) Unique metabolites significantly changed after exercise at ZT3 (B) and ZT15 (C) either in a specific tissue alone or in combination with different tissues. Data placed as a frequent order. Vertical bars indicate set size, horizonal bars indicate intersection size, and dots and lines indicate set identity.

(D and E) Correlation charts displaying metabolites changed only in each specific combination of two different tissues after exercise at ZT3 (D) and ZT15 (E). Correlation coefficient and significance are measured based on the Pearson correlation coefficient. * $p<0.033$ and ***$p<0.001$.

The metabolomic data contain 5-6 individual tissues per group. Two-way ANOVA was applied for the detection of the significance of altered levels of metabolites using p-value cutoff of 0.05.

A

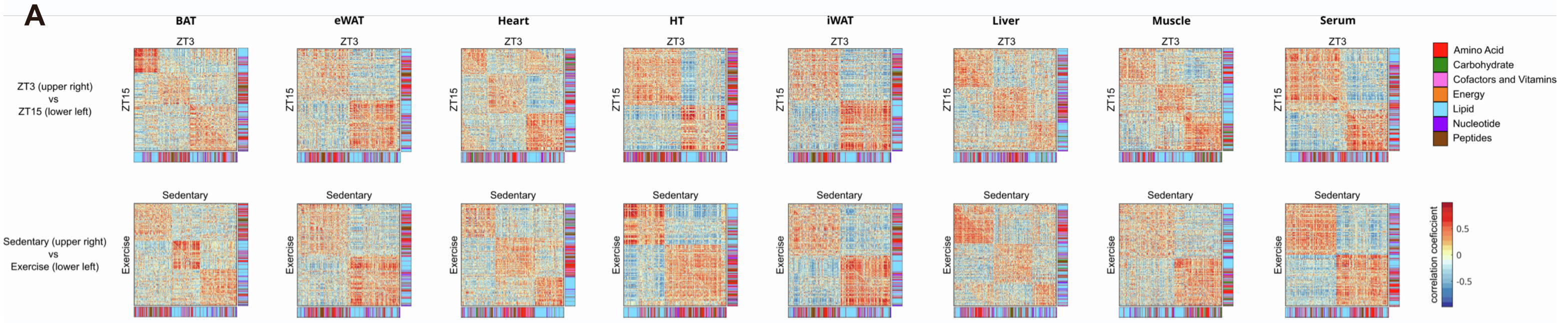

B

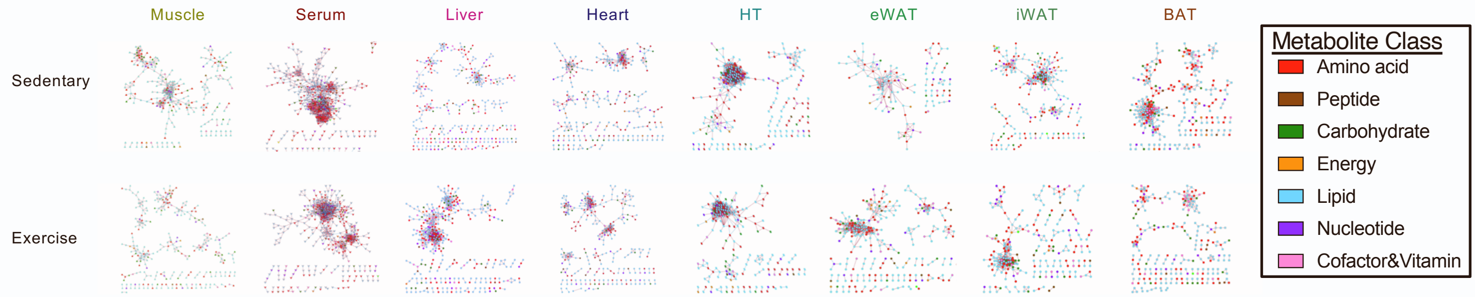

C

tissue	Sedentary Nodes (metabolites)	Exercise Nodes (metabolites)	Sedentary Edges (correlations)	Exercise Edges (correlations)
Muscle	274	293	535	383
Serum	390	391	1321	1120
Liver	344	387	456	1049
Heart	283	294	487	605
HT	236	201	1404	933
eWAT	133	276	225	595
iWAT	251	245	712	573
BAT	288	253	692	334

D

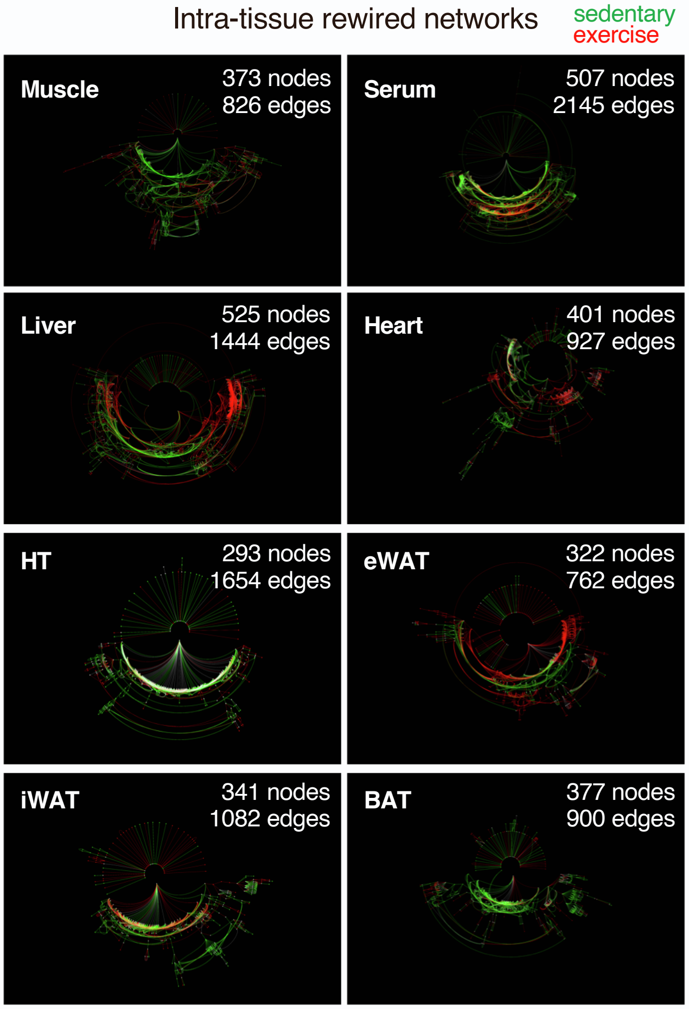

E

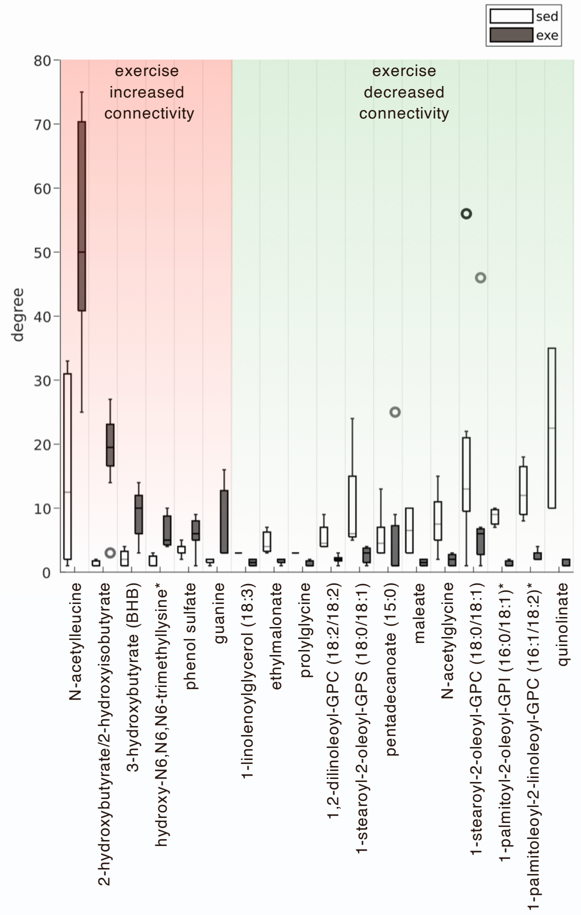

F

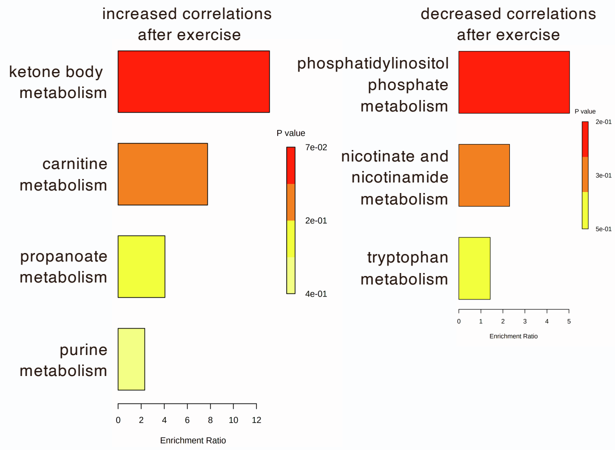

Figure S4 Intra-tissue metabolite correlations (Related to Figure 3)

(A) Correlation heatmaps for metabolites in each tissue. Upper row compares correlations dependent on time ZT15 vs ZT3, lower row compares correlations upon exercise versus sedentary. Metabolite class is indicated on the bottom and right color bars. Correlation coefficient rho is shown as red (positive) or blue (negative) as indicated.

(B) Networks of significantly correlated metabolites detected in each tissue under sedentary or exercise conditions. Each node refers to a metabolite, color refers to metabolite class. Edges are drawn for each significant intra-tissue correlation, and color refers to correlation coefficient sign (red, positive; blue, negative).

(C) Total number of correlated metabolite nodes and edges detected in each tissue.

(D) Combined DyNet visualization of metabolite interaction networks highlighting rewired nodes and edges in each tissue. Red nodes and edges are present only under exercise; green nodes and edges are present only under sedentary conditions; grey nodes and edges are present under both.

(E) Comparison of metabolite degree distributions over all tissues between exercise (gray) and sedentary (white) conditions. A change in degree distribution was estimated using Two-sample Kolmogorov-Smirnov goodness-of-fit hypothesis test. Only metabolites with significantly altered degree distribution are shown. Red background refers to metabolites with an increase in the degree distribution upon exercise, green background to metabolites with decreased distributions.

Horizontal line indicates the median, the size of the boxes refers to upper and lower quartiles, whiskers are minimum and maximum values, and circles refer to outliers.

(F) Bar charts showing Metaboanalyst KEGG pathway enrichment ratio for metabolites that shift degree distribution upon exercise.

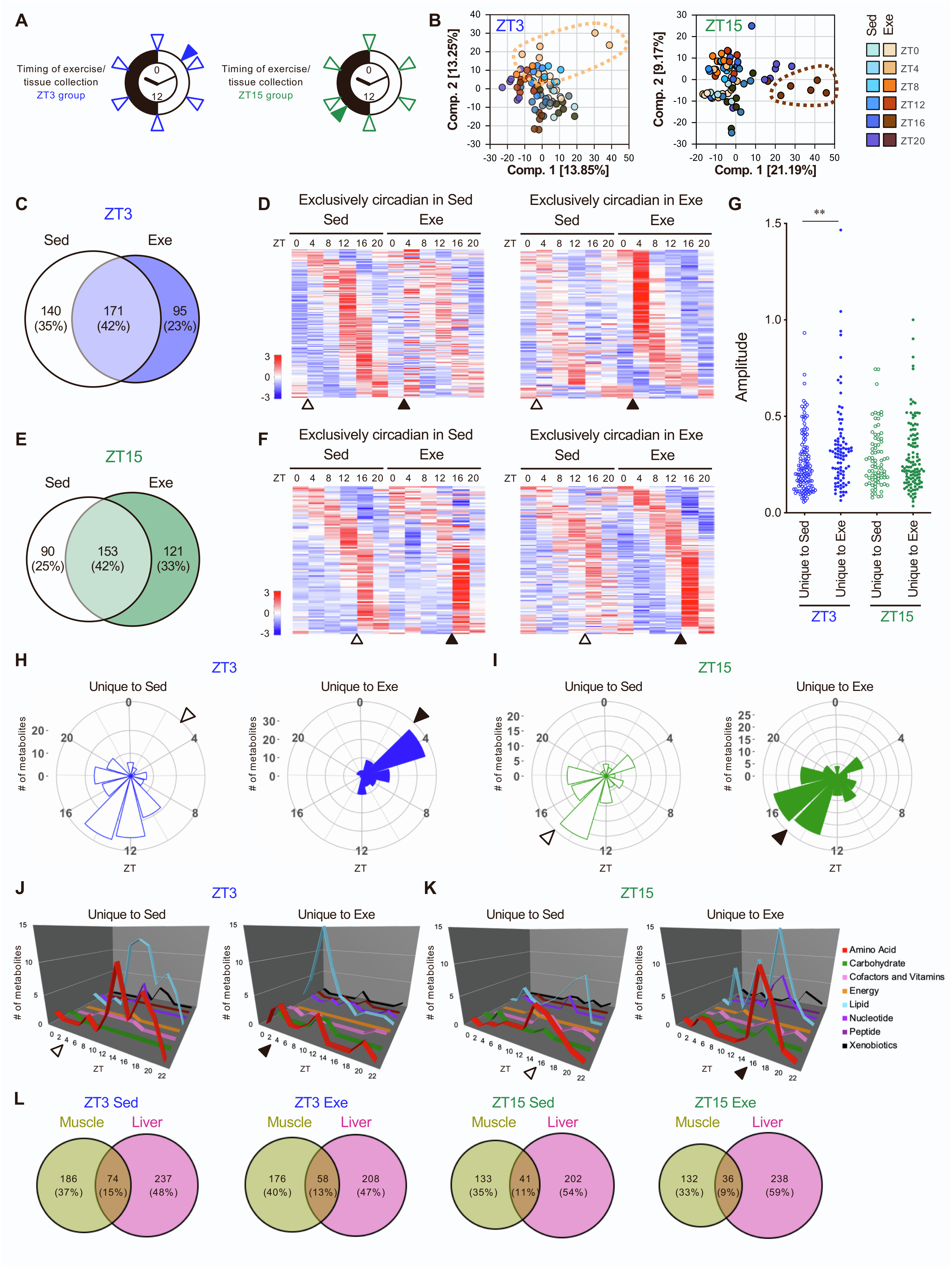
A
Timing of exercise/
tissue collection
ZT3 group
ZT15 group
0
12
B
ZT3
ZT15
Comp. 1 [13.85%]
Comp. 2 [13.25%]
Comp. 1 [21.19%]
Comp. 2 [9.17%]
Sed
Exe
ZT0
ZT4
ZT8
ZT12
ZT16
ZT20
C
ZT3
Sed
Exe
140
(35%)
171
(42%)
95
(23%)
D
Exclusively circadian in Sed
Exclusively circadian in Exe
Sed
Exe
ZT
0 4 8 12 16 20
E
ZT15
90
(25%)
153
(42%)
121
(33%)
F
Exclusively circadian in Sed
Exclusively circadian in Exe
G
Amplitude
**
Unique to Sed
Unique to Exe
ZT3
ZT15
H
ZT3
Unique to Sed
Unique to Exe
# of metabolites
ZT
I
ZT15
Unique to Sed
Unique to Exe
J
ZT3
Unique to Sed
Unique to Exe
K
ZT15
Amino Acid
Carbohydrate
Cofactors and Vitamins
Energy
Lipid
Nucleotide
Peptide
Xenobiotics
L
ZT3 Sed
ZT3 Exe
ZT15 Sed
ZT15 Exe
Muscle
Liver
186
(37%)
74
(15%)
237
(48%)
176
(40%)
58
(13%)
208
(47%)
133
(35%)
41
(11%)
202
(54%)
132
(33%)
36
(9%)
238
(59%)

Figure S5 Liver 24h metabolome under exercise at different times of day (Related to Figure 4)
(A) Daily timing for the acute exercise during the early rest phase (ZT3) and early active phase (ZT15) and tissue collection every 4h after exercise or sham exercise. Clock icon indicates the timing of exercise (filled triangles) and subsequent tissue collection (open triangles).
(B) PCA plots illustrating the distribution of liver metabolites from the sedentary (Sed) mice versus exercise (Exe) mice at corresponding time of day.
(C) Venn diagram showing the number and ratio of cycling metabolites in liver collected from the Sed and Exe mice at the early rest phase (ZT15).
(D) Heatmaps displaying metabolites exclusively circadian in the Sed (left panel) and Exe (right panel) mice at the early rest phase.
(E) The number and ratio of rhythmic metabolites in the Sed liver and Exe liver at the early active phase (ZT15).
(F) Cycling liver metabolites found exclusively in the Sed and Exe mice at the early active phase.
(G) Amplitude distribution of rhythmic metabolites in the Sed and Exe mice at the early rest phase versus the early active phase. **$p<0.01$ analyzed by Student's t-test.
(H and I) The phase lag of circadian metabolites unique to the Sed (left) and Exe (right) mice at the early rest phase (ZT3, H) and the early active phase (ZT15, I).
(J and K) Temporal metabolites landscape of circadian metabolites exclusively in the Sed (left) and Exe (right) liver at the early rest phase (ZT3, J) and the early active phase (ZT15, K).
(L) Overlap of rhythmic metabolites between muscle and liver in 4 different conditions, including the Sed mice at ZT3, Exe mice at ZT3, Sed mice at ZT15, and Exe mice at ZT15. Venn diagrams showing the number and ratio of cycling metabolites either or both in muscle and liver.
Sed; Sedentary, Exe; Exercise.

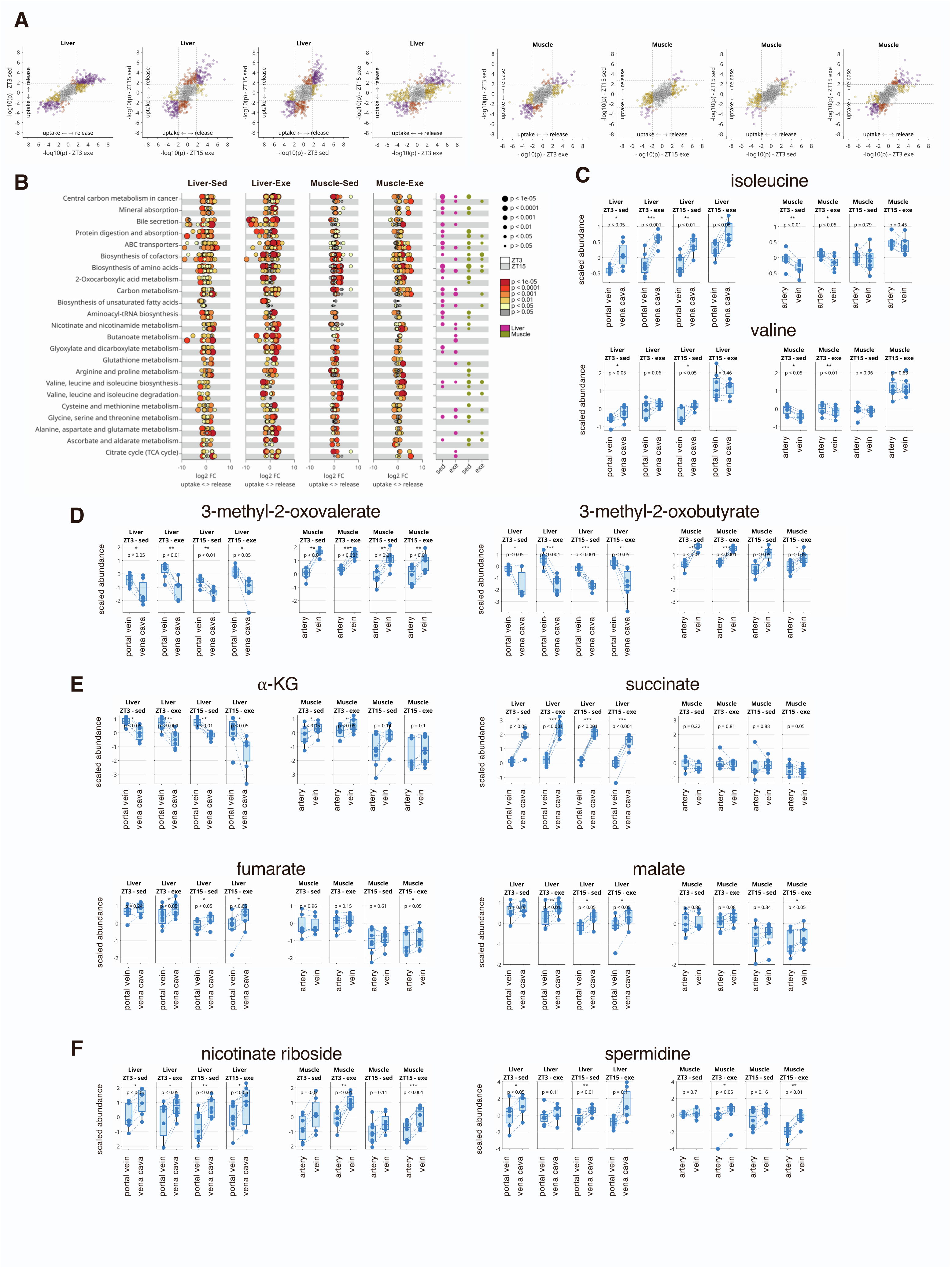

A
Liver
Muscle
-log10(p) - ZT3 sed
-log10(p) - ZT15 sed
-log10(p) - ZT15 exe
-log10(p) - ZT3 exe
-log10(p) - ZT15 exe
-log10(p) - ZT3 sed
uptake ← → release
B
Liver-Sed
Liver-Exe
Muscle-Sed
Muscle-Exe
Central carbon metabolism in cancer
Mineral absorption
Bile secretion
Protein digestion and absorption
ABC transporters
Biosynthesis of cofactors
Biosynthesis of amino acids
2-Oxocarboxylic acid metabolism
Carbon metabolism
Biosynthesis of unsaturated fatty acids
Aminoacyl-tRNA biosynthesis
Nicotinate and nicotinamide metabolism
Butanoate metabolism
Glyoxylate and dicarboxylate metabolism
Glutathione metabolism
Arginine and proline metabolism
Valine, leucine and isoleucine biosynthesis
Valine, leucine and isoleucine degradation
Cysteine and methionine metabolism
Glycine, serine and threonine metabolism
Alanine, aspartate and glutamate metabolism
Ascorbate and aldarate metabolism
Citrate cycle (TCA cycle)
log2 FC
uptake < > release
sed
exe
p < 1e-05
p < 0.0001
p < 0.001
p < 0.01
p < 0.05
p > 0.05
ZT3
ZT15
Liver
Muscle
C
isoleucine
valine
D
3-methyl-2-oxovalerate
3-methyl-2-oxobutyrate
E
α-KG
succinate
fumarate
malate
F
nicotinate riboside
spermidine
scaled abundance
Liver ZT3 - sed
Liver ZT3 - exe
Liver ZT15 - sed
Liver ZT15 - exe
Muscle ZT3 - sed
Muscle ZT3 - exe
Muscle ZT15 - sed
Muscle ZT15 - exe
portal vein
vena cava
artery
vein

Figure S6 Arteriovenous differences of liver and muscle metabolites after timed exercise (Related to Figure 6)
(A) Correlation plot for metabolite uptake/release under sedentary (sed) and exercise (exe) conditions within liver and muscle according to time. Directed p-values are defined as –log10(p) times sign of fold change. Dashed lines denote FDR-adjusted significance alpha level of 0.05. Each dot refers to one metabolite under different conditions. Colors refer to significance assignment. Purple dots are significant in both conditions, yellow dots only in x-axis condition, orange in y-axis, and grey dots are not significant.
(B) KEGG enrichment analysis identifying time-dependent impact of exercise on liver and muscle metabolite uptake/release under sedentary (sed) or exercise (exe) conditions. Tissue-specific metabolite dotplots for liver and muscle under exercise and sedentary conditions show significant metabolites mapped to specific pathways. log2 FC (fold-change) refers to uptake and release. Size and color of the dots indicate significance of regulated (-log10(p)) metabolites. Grey dots refer to metabolites mapped to the pathway, but not significantly regulated. White background areas indicate metabolites at ZT3 and grey background indicates metabolites at ZT15. Significance of whole pathway enrichment is displayed in the right dotplot. Colors of the dots refer to tissue and size refers to the enrichment significance (-log10(p)). Significant ($p<0.01$) pathways are shown.
(C-F) Boxplots and relative A/V differences of selected metabolites (n=6; all adjusted *$p<0.05$, **$p<0.01$, and ***$p<0.001$, paired t-test; horizontal line of boxes indicates the median, size of the boxes reflect upper and lower quartiles, whiskers are minimum and maximum values, and dashed lines connect A/V data from individual mice).

A

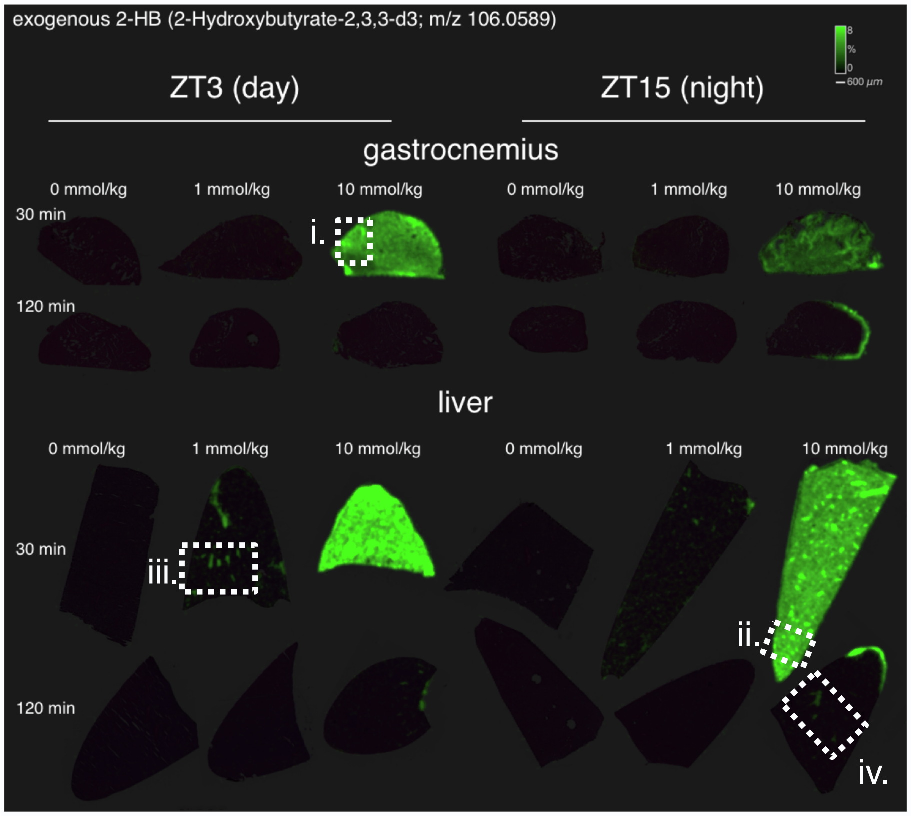

B

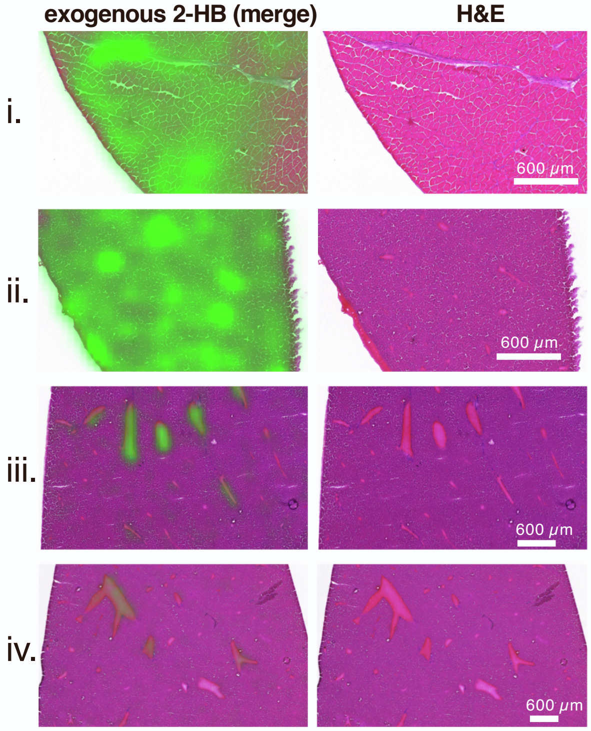

C

metabolites correlated with exogenous 2-HB (30min; adjp<0.05; rho>0.4)

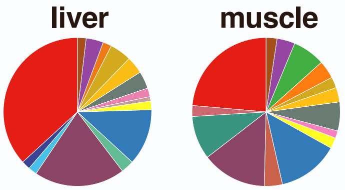

liver (106)	muscle (127)	class
39	30	Amino acids & peptides
21	18	Fatty Acids & Conjugates
13	17	Monosaccharides
0	12	Eicosanoids
0	9	Sterols
4	8	Purines
4	5	TCA acids
0	5	Indoles
2	5	Steroids
5	3	Short-chain acids & derivatives
4	4	Pyrimidines
2	3	Organic dicarboxylic acids
2	3	Tryptamines
0	3	Bile acids
3	0	Imidazoles
2	2	Phenylacetic acids
2	0	Benzoic acids
2	0	Benzamides
1	0	Phenols

liver

enriched KEGG pathway	hits	FDR
Alanine, aspartate & glutamate metabolism	13	0.000
Arginine biosynthesis	9	0.000
Aminoacyl-tRNA biosynthesis	17	0.000
Arginine & proline metabolism	14	0.001
Valine, leucine & isoleucine biosynthesis	6	0.001
D-Glutamine & D-glutamate metabolism	5	0.001
Histidine metabolism	8	0.002
Fructose & mannose metabolism	9	0.002
beta-Alanine metabolism	9	0.002
Pantothenate & CoA biosynthesis	8	0.004
Glycine, serine & threonine metabolism	11	0.004
Butanoate metabolism	7	0.004
Ascorbate & aldarate metabolism	5	0.005
Glycolysis / Gluconeogenesis	9	0.009
Pyruvate metabolism	8	0.010
Galactose metabolism	9	0.010
Pentose & glucuronate interconversions	7	0.012
Phenylalanine metabolism	5	0.013
Tryptophan metabolism	11	0.020
Phenylalanine, tyrosine & tryptophan biosynthesis	3	0.021
D-Arginine and D-ornithine metabolism	3	0.021
Pentose phosphate pathway	7	0.032
Pyrimidine metabolism	10	0.034
Valine, leucine & isoleucine degradation	10	0.039

muscle

enriched KEGG pathway	hits	FDR
Pentose phosphate pathway	14	0.000
Tryptophan metabolism	19	0.000
Fructose & mannose metabolism	11	0.000
Arginine & proline metabolism	15	0.001
Galactose metabolism	11	0.007
Alanine, aspartate & glutamate metabolism	11	0.009
beta-Alanine metabolism	9	0.011
Ascorbate & aldarate metabolism	5	0.019
Pantothenate & CoA biosynthesis	8	0.020
Amino sugar & nucleotide sugar metabolism	12	0.021
Histidine metabolism	7	0.024
Glycolysis / Gluconeogenesis	9	0.037
Arginine biosynthesis	6	0.045

D

top 20 metabolites +/- correlated with exogenous 2-HB (30 min & 120 min)

liver

Compound name	m/z	adduct	rho	FDR
(S)-Lactate	89.0243	M-H	0.79	0.0E+00
L-Alanine	88.0403	M-H	0.71	0.0E+00
4-Aminobutyraldehyde	108.0431	M+Na-2H	0.70	1.8E-14
Guanidinoacetate	98.0355	M-H2O-H	0.69	1.8E-14
3,4-Dihydroxy-L-phenylalanine	178.051	M-H2O-H	0.68	2.0E-178
D-Glucuronolactone	175.0248	M-H	0.65	0.0E+00
L-Histidine	154.0621	M-H	0.64	0.0E+00
L-Glutamate	146.0458	M-H	0.63	0.0E+00
D-Glucose	215.0323	M+Cl	0.63	1.2E-69
3-Hydroxyanthranilate	174.017	M+Na-2H	0.62	0.0E+00
Adenine	134.0472	M-H	-0.48	6.0E-76
L-Glutamine	145.0618	M-H	-0.49	2.3E-06
7α-Hydroxycholest-4-en-3-one	435.3026	M+Cl	-0.50	0.0E+00
AMP	346.0566	M-H	-0.50	1.3E-84
Guanine	150.042	M-H	-0.51	5.2E-86
UMP	323.0288	M-H	-0.51	1.3E-86
Tryptamine	159.0926	M-H	-0.52	2.8E-07
L-Phenylalanine	146.0612	M-H2O-H	-0.54	1.3E-07
Indole-3-acetaldehyde	158.0612	M-H	-0.56	2.7E-08
Serotonin	157.0771	M-H2O-H	-0.78	7.5E-21

muscle

Compound name	m/z	adduct	rho	FDR
4-Aminobutyraldehyde	108.0431	M+Na-2H	0.65	2.5E-76
Thymidine	277.0589	M+Cl	0.64	1.8E-73
Guanidinoacetate	98.0355	M-H2O-H	0.63	6.3E-49
D-Glucuronolactone	175.0248	M-H	0.63	0.0E+00
Pantetheine	277.1232	M-H	0.63	3.3E-48
Glutathione	344.0331	M+K-2H	0.62	9.3E-70
(S)-Lactate	89.0243	M-H	0.62	0.0E+00
Cytidine	264.0596	M+Na-2H	0.61	6.3E-67
Hexadecanoic acid	255.2331	M-H	0.61	1.2E-66
D-Glucose 6-phosphate	259.0225	M-H	0.61	2.9E-66
L-2-Amino-3-oxobutanoic acid	152.0115	M+Cl	-0.58	7.7E-04
L-Proline	152.0115	M+K-2H	-0.58	7.7E-04
Octanoic acid	143.1076	M-H	-0.59	6.6E-05
L-Serine	140.0118	M+Cl	-0.59	6.0E-05
L-Glutamate	146.0458	M-H	-0.60	3.2E-05
4-Hydroxybenzoate	137.0244	M-H	-0.64	6.7E-05
(S)-Malate	133.0142	M-H	-0.64	5.3E-06
L-Leucine	130.0872	M-H	-0.65	5.0E-06
L-Aspartate	132.0302	M-H	-0.77	1.5E-09
Serotonin	157.0771	M-H2O-H	-0.84	3.0E-11

Figure S7 Uptake and impact of exogenous 2-HB on liver and muscle metabolism (Related to Figure 7)
(A) Mouse muscle (gastrocnemius) and liver sections after MALDI imaging mass spectrometry. Green signal intensity indicates relative tissue levels of exogenous 2-HB (green) after subcutaneous administration of different doses (0, 1, or 10 mmol/kg), at different durations (30 min or 120 min) and at different times of day (ZT3 or ZT15).
(B) Zoomed regions from panel (A), along with underlying hematoxylin and eosin staining (H&E).
(C) Left pie charts and table of tissue metabolites significantly correlated with exogenous 2-HB 30 min after administration (Benjamini-Hochberg adjusted $p<0.05$, Spearman coefficient > 0.4 or < -0.4). Middle and right tables show Metaboanalyst KEGG pathway enrichment of same metabolites.
(D) Table of top 20 significantly positive/negative exogenous 2-HB-associated metabolites 30 min and 120 min after administration (Benjamini-Hochberg adjusted $p<0.05$, Spearman coefficient > 0.4 or < -0.4).
