# Supplementary figures and images for: Atlas of exercise metabolism reveals time-dependent signatures of metabolic homeostasis
Source: Cell Metab. Author manuscript; Available in PMC 2026 May 20. (PMC13189211; doi:10.1016/j.cmet.2021.12.016)

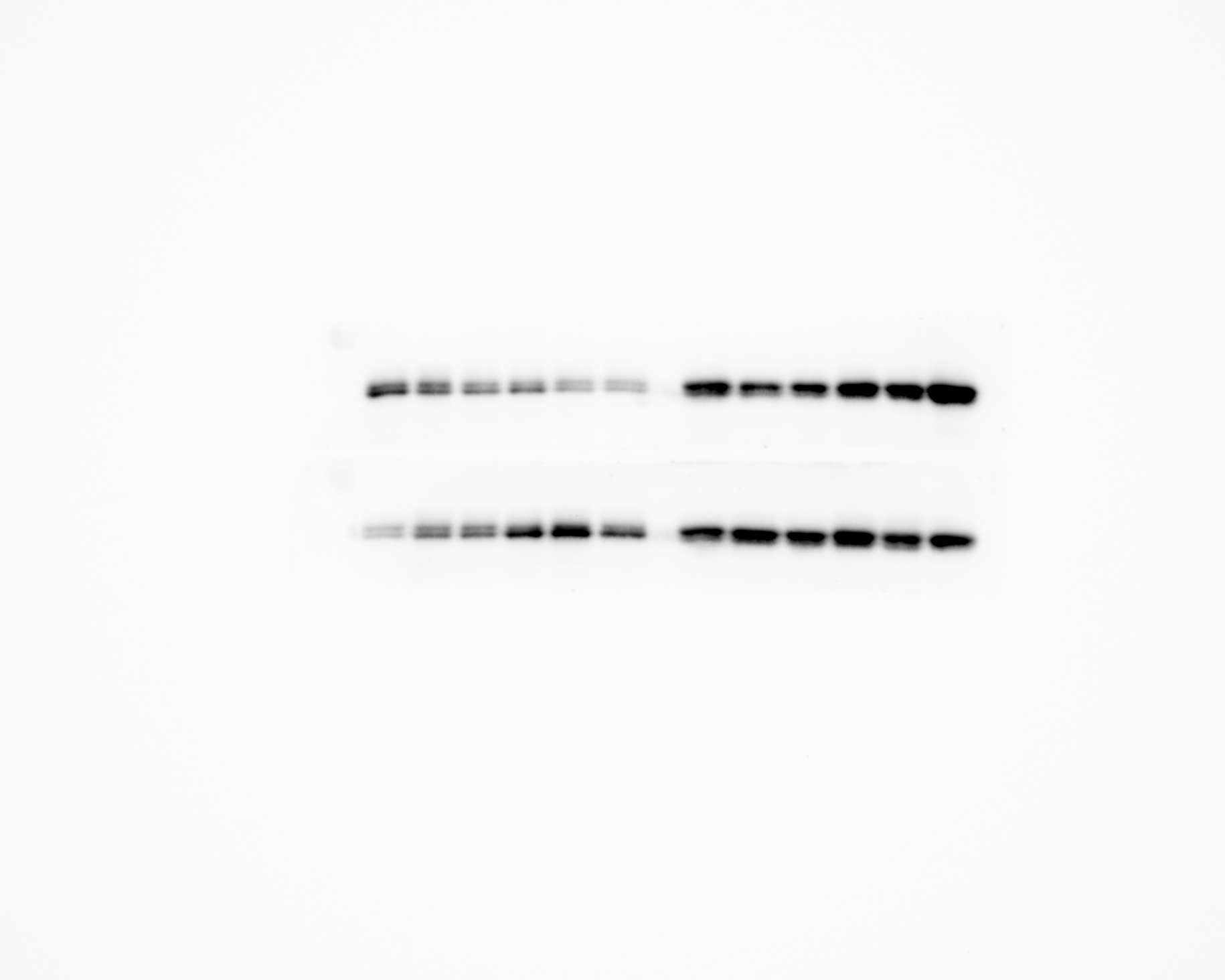

Supplement: Data_S1 [file NIHMS2168542-supplement-Data_S1.zip › Data S1_Original datasource used to create all graphs and uncropped western blot images (Related to Figure 1-7)/Orig Data Fig 5D_P-AMPK_Muscle_Liver.tif]

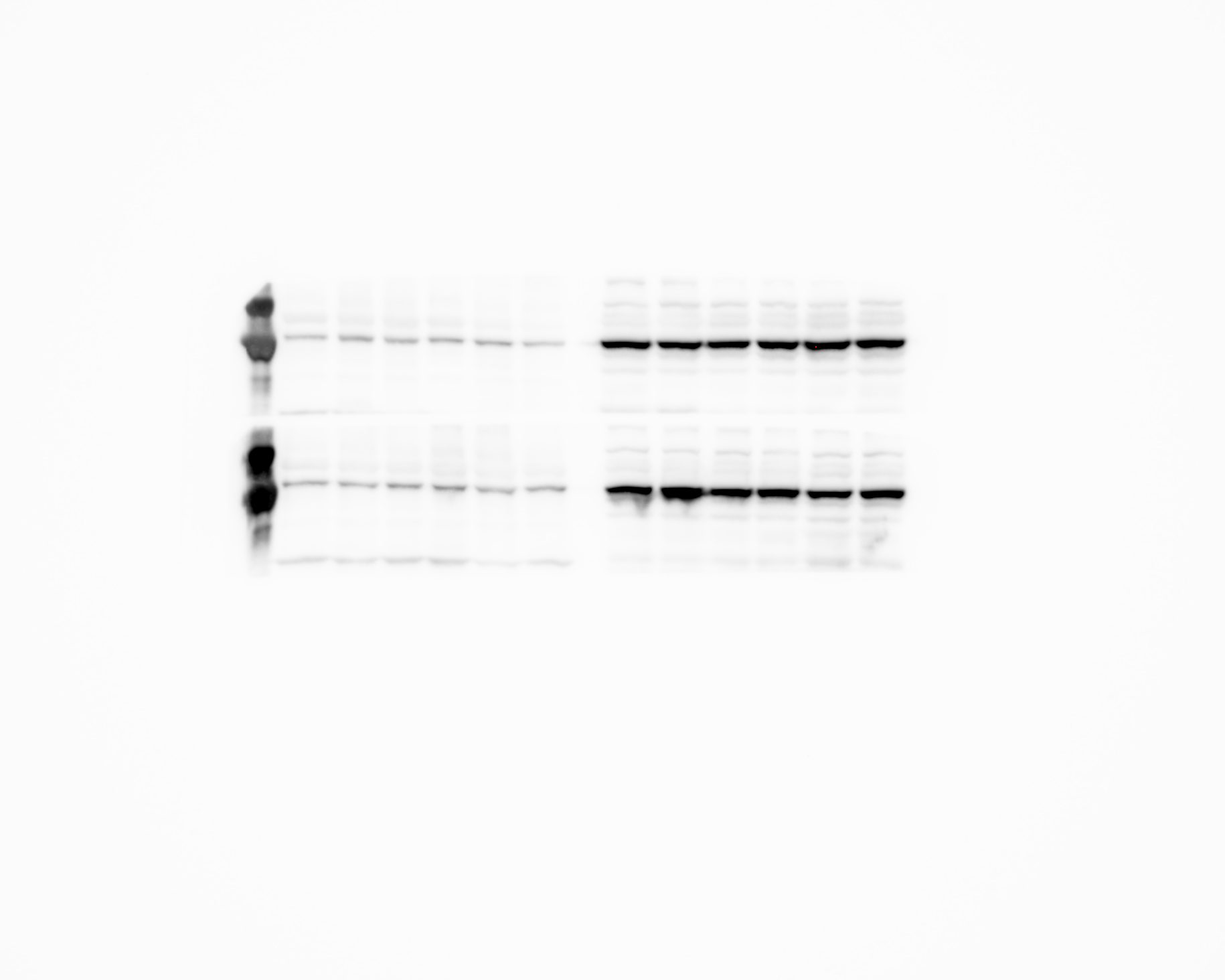

Supplement: Data_S1 [file NIHMS2168542-supplement-Data_S1.zip › Data S1_Original datasource used to create all graphs and uncropped western blot images (Related to Figure 1-7)/Orig Data Fig 5D_p84_Muscle_Liver.tif]

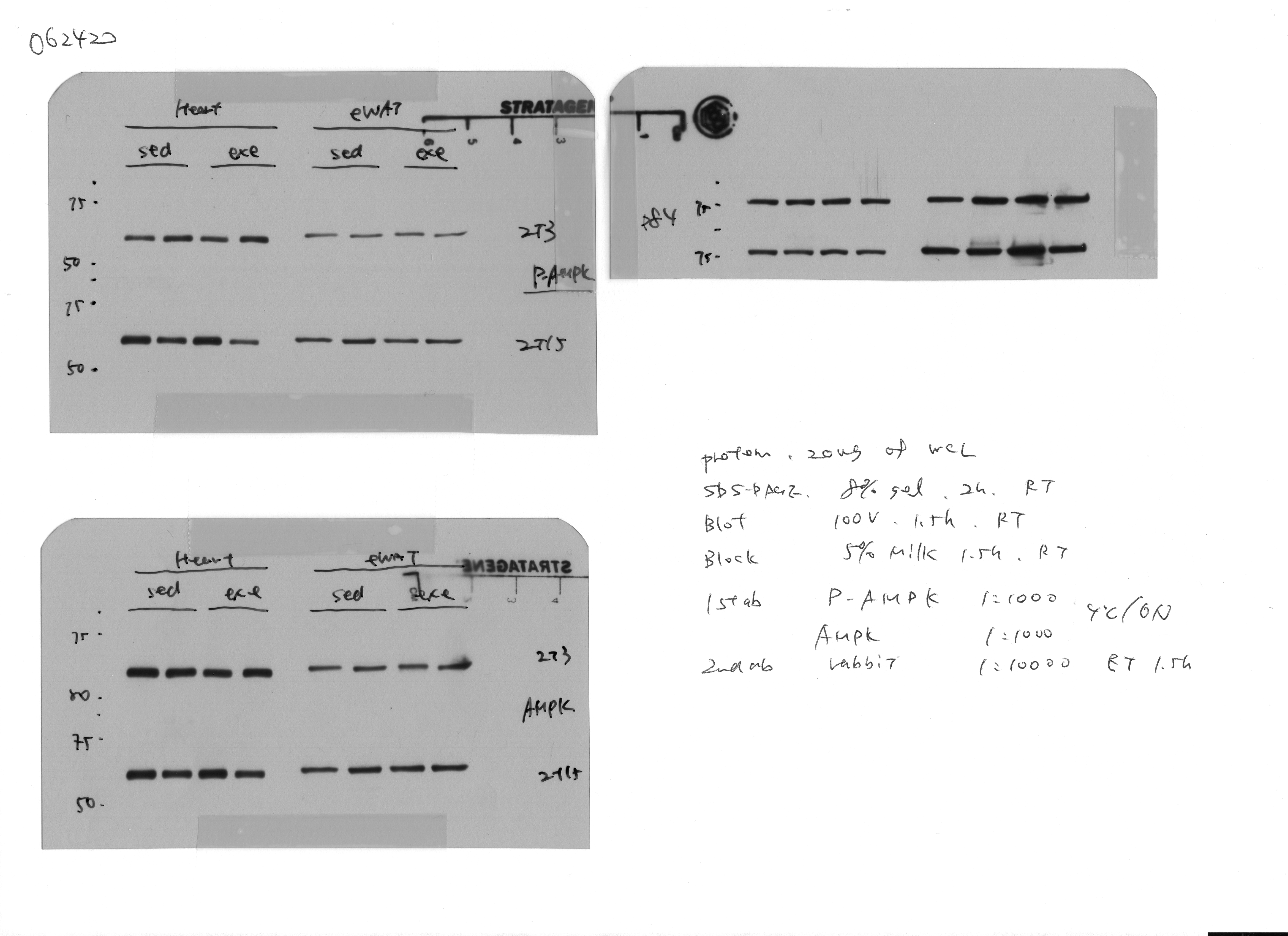

Supplement: Data_S1 [file NIHMS2168542-supplement-Data_S1.zip › Data S1_Original datasource used to create all graphs and uncropped western blot images (Related to Figure 1-7)/Orig Data Fig 5D_P-AMPK_AMPK_p84_Heart_WAT.tif]

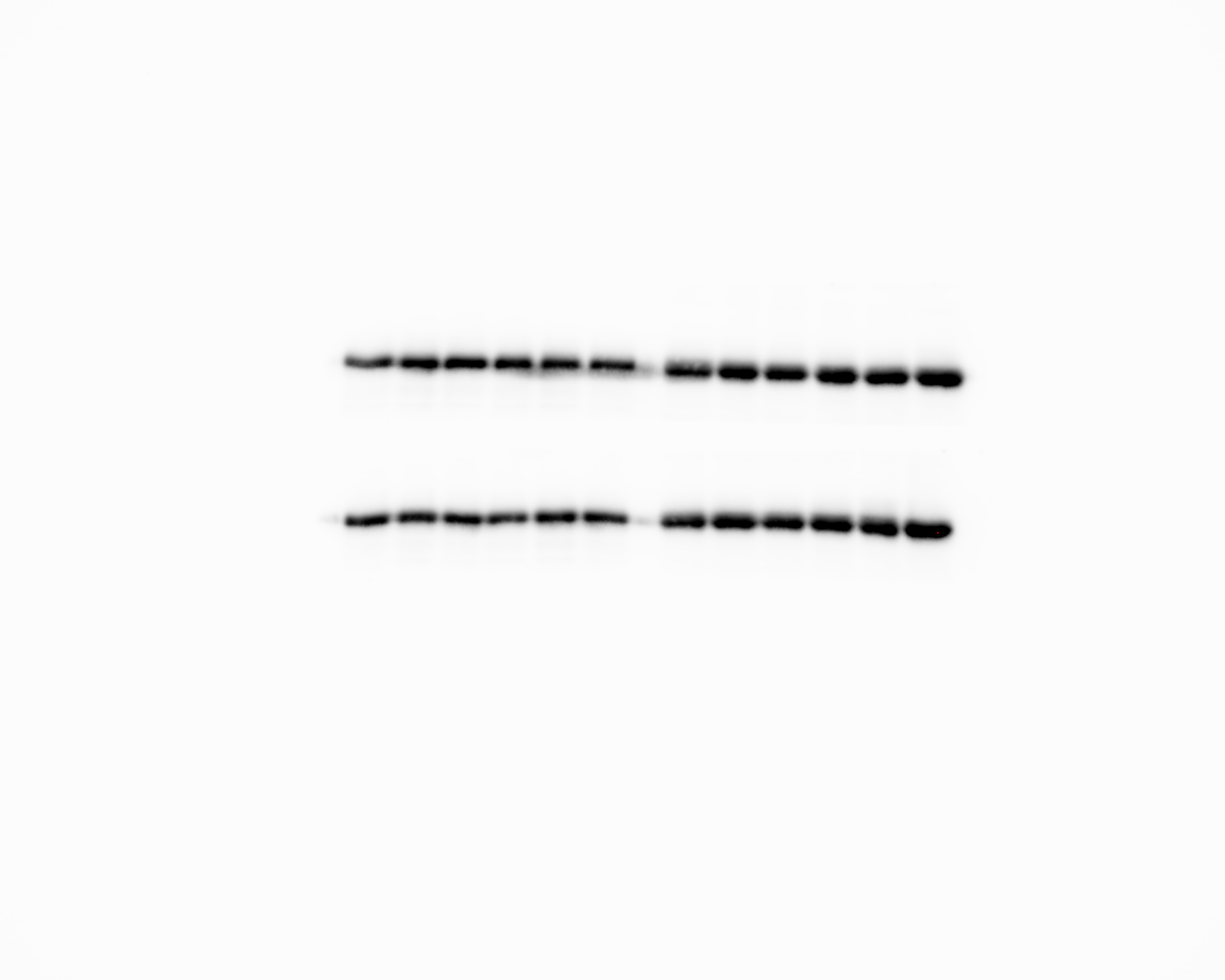

Supplement: Data_S1 [file NIHMS2168542-supplement-Data_S1.zip › Data S1_Original datasource used to create all graphs and uncropped western blot images (Related to Figure 1-7)/Orig Data Fig 5D_AMPK_Muscle_Liver.tif]
